# Copper-promoted C5-selective bromination of 8-aminoquinoline amides with alkyl bromides

**DOI:** 10.3762/bjoc.20.14

**Published:** 2024-01-23

**Authors:** Changdong Shao, Chen Ma, Li Li, Jingyi Liu, Yanan Shen, Chen Chen, Qionglin Yang, Tianyi Xu, Zhengsong Hu, Yuhe Kan, Tingting Zhang

**Affiliations:** 1 Jiangsu Provincial Key Laboratory for Chemistry of Low-Dimensional Materials, School of Chemistry and Chemical Engineering, Huaiyin Normal University, Huai'an, 223300, Jiangsu, China

**Keywords:** aminoquinolines, C–H bromination, copper catalysis, regioselectivity

## Abstract

An efficient and practical method for the synthesis of C5-brominated 8-aminoquinoline amides via a copper-promoted selective bromination of 8-aminoquinoline amides with alkyl bromides was developed. The reaction proceeds smoothly in dimethyl sulfoxide (DMSO) under air, employing activated and unactivated alkyl bromides as the halogenation reagents without additional external oxidants. This method features outstanding site selectivity, broad substrate scope, and excellent yields.

## Introduction

Over the past decades, the 8-aminoquinoline motif that could be found in several natural products [1] has attracted significant attention for its widespread usage in pharmaceuticals [2], agrochemicals [3], and functional materials [4].

In particular, the aminoquinoline scaffold has emerged as an important auxiliary group for the proximal C–H activation with the efforts of Daugulis [5] and others [6]. Results from medical research indicated that the introduction of halogen atoms into quinoline motifs has positive effects on their bioactivities, such as antimalarial, antitumor, and so on [7]. Therefore, it is of great importance to develop syntheses for 8-aminoquinoline derivatives with diverse substituent groups, especially those leading to halogenated derivatives.

**Scheme 1:**
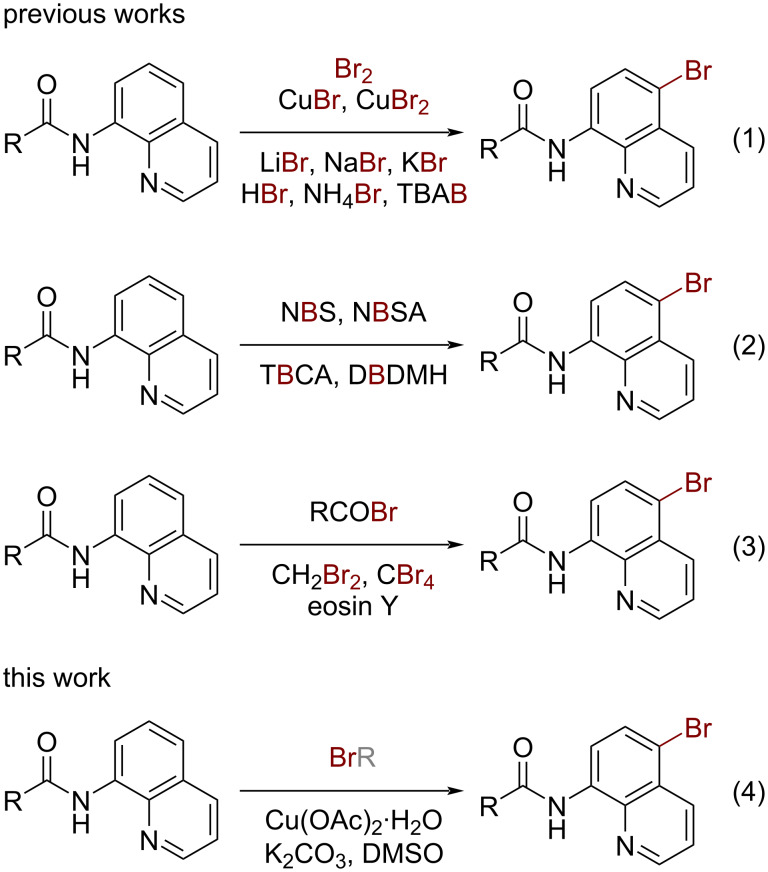
Methods for the C5-selective bromination of 8-aminoquinoline amides.

Recent years have witnessed much progress in halogenation reactions of the quinoline ring in the C2–C7 positions [8–9]. Among them, the synthesis of important C5-halogenated products gained particular attention since Stahl et al. reported the first chlorination example using LiCl as the halogen source [10]. Following this pioneering work, elegant strategies for the C5–H bromination of the quinoline ring employing simple inorganic and organic bromine-containing compounds such as Br_2_, CuBr, CuBr_2_, LiBr, NaBr, KBr, HBr, NH_4_Br, and tetrabutylammonium bromide (TBAB) as halogen sources have been realized (Scheme 1, reaction 1) [11–20]. Another category of extensively used bromination reagents are brominated imides, such as *N*-bromosuccinimide (NBS), *N*-bromosaccharin (NBSA), tribromoisocyanuric acid (TBCA), 1,3-dibromo-5,5-dimethylhydantoin (DBDMH), and so on (Scheme 1, reaction 2) [21–24]. However, reports on the bromination of C5–H of 8-aminoquinolines employing acyl bromides, alkyl bromides, and aryl bromides as bromination reagents are limited. Wan and Li, respectively, demonstrated a few examples of a one-pot *N*-acylation and C5–H bromination of 8‑aminoquinolines using acyl bromines acting as both acyl and halide donors [25–26]. The groups of Lei and Fang independently realized the selective C5-bromination of 8-aminoquinoline amides using carbon tetrabromide and dibromomethane under photo- and electrocatalysis conditions [27–28]. In 2017, Xia and co-workers reported a novel, mild, metal-free, and regioselective bromination of amides, wherein the organic dye eosin Y acted as the bromine source in combination with Selectfluor^®^ (Scheme 1, reaction 3) [29].

Despite significant progress in this area, most of these methodologies still suffer from the use of external oxidants, complex reaction equipment, and expensive and/or toxic halogen sources, which limit the practicality for large-scale use. Therefore, novel bromination reagents and simple bromination approaches are still required to be established. Very recently, we reported a copper-catalyzed C5-bromination and difluoromethylation reaction of 8-aminoquinolines using ethyl bromodifluoroacetate as bifunctional reagent [30] and the feasibility of using activated alkyl bromides as bromination reagents has been preliminarily demonstrated. To further expand the scope of bromination reagents and enhance the utility of the reaction method, herein, we wish to report a simple and efficient copper-promoted C5-selective bromination reaction of 8-aminoquinoline amides using activated and unactivated alkyl bromides as the bromine source (Scheme 1, reaction 4).

## Results and Discussion

At the beginning of this investigation, *N*-(quinolin-8-yl)benzamide (**1a**) and ethyl bromoacetate (**2a**) were selected as model substrates to screen the reaction parameters (Table 1). The treatment of **1a** with **2a** (4.0 equiv) in the presence of FeCl_3_ (20 mol %) and K_3_PO_4_ (1.0 equiv) in DMSO at 100 °C for 12 h gave the brominated product **3aa** in 65% yield (Table 1, entry 1). The bromination was found to selectively take place at the C5-position of the quinoline ring of **1a** in this reaction. Other competitive site-selective C–H bromination products and multiple brominated products were not observed. Subsequently, the bromination reaction was examined with various catalysts such as CoCl_2_·6H_2_O, Ni(OAc)_2_·4H_2_O, MnSO_4_·H_2_O, CuCl, CuBr, CuCl_2_, CuBr_2_, and Cu(OAc)_2_·H_2_O (Table 1, entries 2–9). To our delight, copper salts were effective, giving the desired product **3aa** in excellent yields of 88–95% (Table 1, entries 7–9). Cuprous salts, cobalt chloride, and nickel acetate were partially efficient for the reaction, providing product **3aa** in 85% yield (Table 1, entries 3–6). The catalytic efficiency of MnSO_4_·H_2_O was consistent with FeCl_3_, affording **3aa** in 64% yield (Table 1, entry 2). The reaction was further examined with a series of bases such as Li_2_CO_3_, Na_2_CO_3_, K_2_CO_3_, Cs_2_CO_3_, K_2_HPO_4_, KHCO_3_, KH_2_PO_4_, and KOAc, and the results demonstrated that the reaction proceeds under alkaline conditions (Table 1, entries 10–17). Among the bases screened, K_2_CO_3_ was the most effective one, yielding the desired product **3aa** quantitatively (Table 1, entry 12). Other bases, such as Li_2_CO_3_, Na_2_CO_3_, Cs_2_CO_3_, and K_2_HPO_4_, were also very efficient, giving the product in excellent yields (Table 1, entries 10, 11, 13, and 14). The other bases were found to be less effective (Table 1, entries 15–17). Notably, the bromination reaction was still efficient with a lower catalyst loading (10 mol %) and lower base loading (0.5 equiv), respectively (Table 1, entries 18 and 19). However, the amount of alkyl bromide and temperature affected the reaction significantly (Table 1, entries 20 and 21). Finally, control experiments demonstrated that copper promoted the transformation and a base was the indispensable factor for the reaction (Table 1, entries 22–24). Therefore, a facile and highly efficient C5-bromination protocol has been established.

**Table 1:** Optimization of the reaction conditions for the copper-promoted C5-bromination^a^.

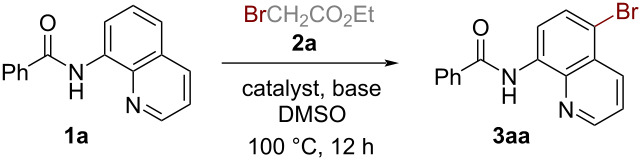			

Entry	Catalyst	Base	Yield (%)^b^

1	FeCl_3_	K_3_PO_4_	65
2	MnSO_4_·H_2_O	K_3_PO_4_	64
3	CoCl_2_·6H_2_O	K_3_PO_4_	85
4	Ni(OAc)_2_·4H_2_O	K_3_PO_4_	85
5	CuCl	K_3_PO_4_	85
6	CuBr	K_3_PO_4_	85
7	CuCl_2_	K_3_PO_4_	92
8	CuBr_2_	K_3_PO_4_	88
9	Cu(OAc)_2_·H_2_O	K_3_PO_4_	95
10	Cu(OAc)_2_·H_2_O	Li_2_CO_3_	93
11	Cu(OAc)_2_·H_2_O	Na_2_CO_3_	97
**12**	**Cu(OAc)** **_2_** **·H** **_2_** **O**	**K** **_2_** **CO** **_3_**	**100 (99)**
13	Cu(OAc)_2_·H_2_O	Cs_2_CO_3_	96
14	Cu(OAc)_2_·H_2_O	K_2_HPO_4_	96
15	Cu(OAc)_2_·H_2_O	KHCO_3_	84
16	Cu(OAc)_2_·H_2_O	KH_2_PO_4_	70
17	Cu(OAc)_2_·H_2_O	KOAc	56
18^c^	Cu(OAc)_2_·H_2_O	K_2_CO_3_	93
19^d^	Cu(OAc)_2_·H_2_O	K_2_CO_3_	94
20^e^	Cu(OAc)_2_·H_2_O	K_2_CO_3_	76
21^f^	Cu(OAc)_2_·H_2_O	K_2_CO_3_	31
22	–	K_3_PO_4_	52
23	Cu(OAc)_2_·H_2_O	–	–
24	–	–	–

^a^Reaction conditions: **1a** (0.2 mmol), **2a** (0.8 mmol), catalyst (20 mol %), base (0.2 mmol), DMSO (1.0 mL), stirred under air in a 35 mL sealed tube. ^b1^H NMR yield with dibromomethane as the internal standard, isolated yield in parentheses. ^c^Catalyst loading was 10 mol %. ^d^0.1 mmol K_2_CO_3_ was used. ^e^0.6 mmol **2a** was used. ^f^Stirred at 80 °C.

**Scheme 2:**
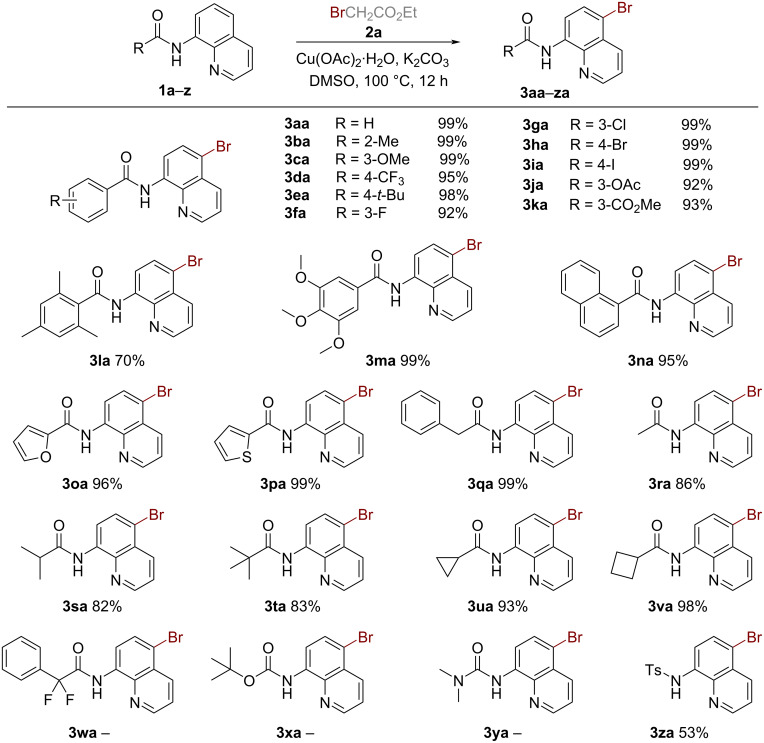
Substrate scope of the 8-aminoquinoline amides. Reaction conditions: **1** (0.2 mmol), **2a** (0.8 mmol), Cu(OAc)_2_·H_2_O (20 mol %), K_2_CO_3_ (0.2 mmol), DMSO (1.0 mL), stirred under air at 100 ºC for 12 h. Isolated yield.

Having identified the optimal reaction conditions for the bromination of *N*-(quinolin-8-yl)benzamide (**1a**) with ethyl bromoacetate (**2a**) (Table 1, entry 12), we next examined the substrate scope and limitations of our method with an array of 8-aminoquinoline amides, and the results are illustrated in Scheme 2. A wide variety of 8-aminoquinoline amides with different substitutions efficiently participated in this mild and versatile bromination. Benzamides with both electron-donating groups (**3ba**–**ca**) and electron-withdrawing groups (**3da**–**fa**) were well tolerated, affording the desired products in excellent yields (92‒99%). These results indicated that the electronic effect of substituents affected the reaction only slightly. Products with substituents with derivatization feasibilities such as halogen (**3ga**–**ia**), acetyloxy (**3ja**), and ester groups (**3ka**) also were obtained in high yields (92‒99%), which demonstrated the practical value of this methodology. Moreover, the reaction showed a good tolerance to sterically hindered substrates like trimethylbenzamide, affording the corresponding brominated product in 70% yield (**3la**). Also the substrate derived from trimethylgallic acid bearing multiple methoxy groups afforded the desired product **3ma** in excellent yield (99%). Noteworthy to mention, substrates containing naphthalene, furan, and thiophene rings also efficiently underwent reaction leading to the quinoline C5-brominated products in excellent yields (**3na**–**pa**). In contrast, side products, substituted on the naphthalene, furan, or thiophene ring were not detected. This protocol was also compatible with linear, branched, and cyclic aliphatic acid-derived substrates (**3qa**–**va**). Surprisingly, small ring-containing substrates displaying significant ring strain were stable under the reaction conditions and afforded the bromination products **3ua** and **3va** in 93 and 98% yield. Finally, some other acyl motifs were investigated (**3wa**–**za**) and the results showed that the protocol could be successfully applied to sulfonamides, albeit giving the target product **3za** in only 53% yield.

**Scheme 3:**
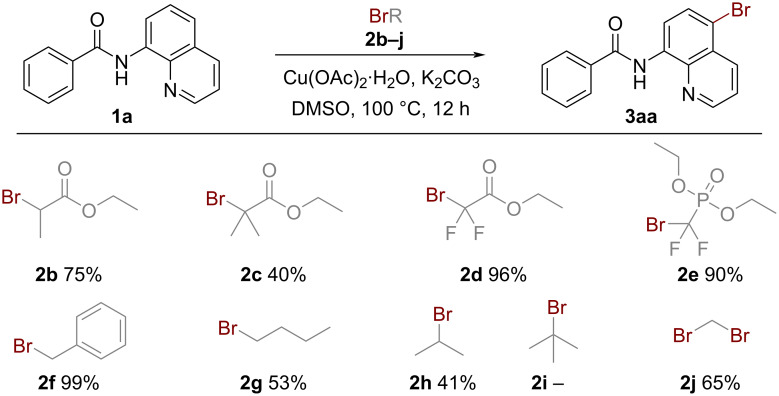
Substrate scope of the bromoalkanes. Reaction conditions: **1a** (0.2 mmol), **2** (0.8 mmol), Cu(OAc)_2_·H_2_O (20 mol %), K_2_CO_3_ (0.2 mmol), DMSO (1.0 mL), stirred under air at 100 ºC for 12 h. Isolated yield.

The scope of the bromination reaction was further extended with various alkyl bromides. As shown in Scheme 3, a series of activated alkyl bromides containing ester, difluoromethylene, benzyl motifs (**2b**–**f**), and unactivated alkyl bromides (**2g**–**i**) were evaluated in this reaction. Activated alkyl bromides such as **2d**, **2e**, and **2f** performed well, affording the brominated product **3aa** in excellent yields (90–99%). However, transformations of amide **1a** to the brominated product **3aa** employing unactivated alkyl bromides (**2g**–**i**) as reaction partners proceeded with low efficiency (0–53%). Notably, the reactivity of primary alkyl bromides is higher than that of secondary alkyl bromides, while the reactivity of tertiary alkyl bromides is the lowest (**2a**–**c, 2g**–**i**). Finally, dibromomethane (**2j**) proceeded well in the reaction, furnishing **3aa** in 65% yield.

**Scheme 4:**
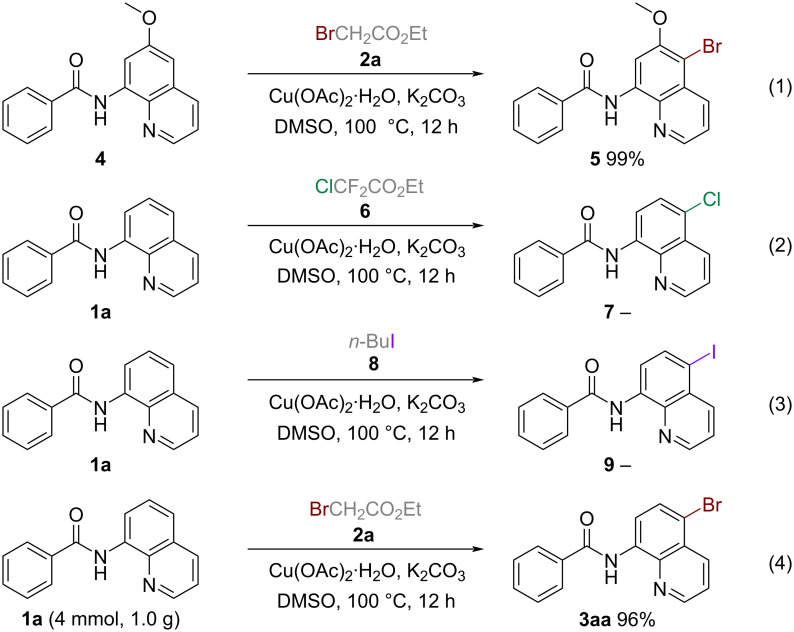
Further substrate scope investigations and gram-scale application.

As showcased in Scheme 4, this methodology is also applicable to substrates containing substituents on the quinoline ring. For example, the 6-OMe-substituted quinoline derivative *N*-(6-methoxyquinolin-8-yl)benzamide (**4**) underwent bromination at the C5 position to give **5** in nearly quantitative yield (Scheme 4, reaction 1). Subsequently, we attempted to apply this method to chlorination and iodination reactions using ethyl chlorodifluoroacetate (**6**) and 1-iodobutane (**8**) as the respective halogenation reagents. However, these attempts ended in failure (Scheme 4, reactions 2 and 3). Furthermore, to demonstrate the synthetic usefulness of the protocol for industrial production, a gram-scale preparation was carried out using **1a**, that afforded the desired product in 96% yield (Scheme 4, reaction 4).

**Scheme 5:**
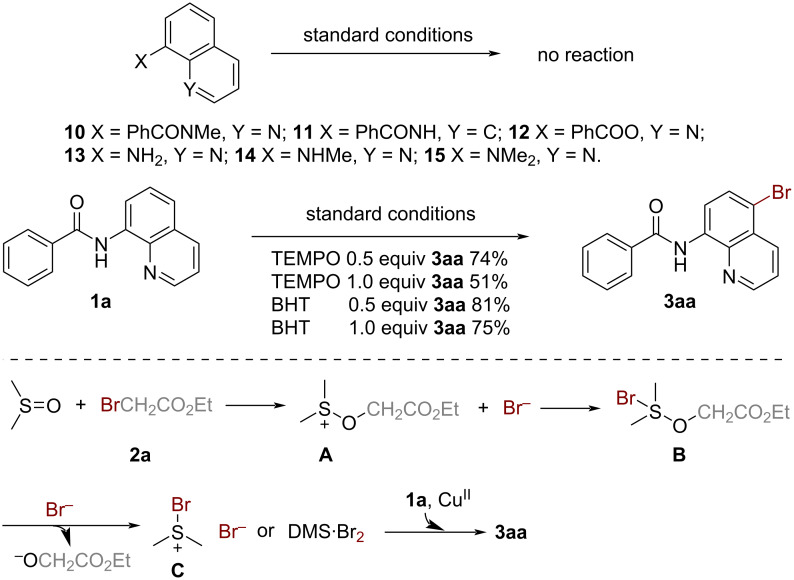
Control experiments and proposed mechanism.

To gain more insight into the reaction mechanism, several control experiments were carried out (Scheme 5). On one hand, the failure of substrates **10**–**15** to participate in the reaction indicated that both the acylamino and quinoline N motifs played a significant role. On the other hand, the stoichiometric amount of free radical inhibitors, including TEMPO and BHT, could not comprehensively suppress the reaction. Based on these experimental results and previous works [30–32], a probable mechanism is proposed. As shown in Scheme 5, ethyl bromoacetate (**2a**) undergoes attack by the dipolar aprotic solvent DMSO to afford the intermediate **A**. This intermediate then reacts with the bromine anion to give intermediate **B**. Dimethylsulfonium bromide or dimethyl thioether/molecular bromine intermediate **C** is then generated, followed by the combination of the bromine anion with intermediate **B**. Finally, selective C5 bromination is accomplished via aromatic electrophilic substitution of **1a** with intermediate **C** promoted by the copper catalyst to afford the desired product **3aa**.

## Conclusion

In summary, we have developed an efficient and practical method for the synthesis of C5-brominated 8-aminoquinoline amides in good to excellent yields via a copper-promoted selective bromination using alkyl bromides as the bromide source. This methodology is scalable, proceeds well with various aromatic and aliphatic amide substrates, and proceeds well with activated and unactivated alkyl bromides. Further studies on mechanistic details and the persistent exploration of halogen sources are ongoing in our laboratory.

## Experimental

A 35 mL sealed tube equipped with a stirring bar was charged with 8-amidequinolines (**1**, 0.2 mmol, 1.0 equiv), BrR (**2**, 0.8 mmol, 4.0 equiv), Cu(OAc)_2_·H_2_O (8.0 mg, 0.04 mmol, 20 mol %), K_2_CO_3_ (27.6 mg, 0.2 mmol, 1.0 equiv), and DMSO (1.0 mL). The tube was sealed with a Teflon cap under air, then the mixture was stirred at 100 ºC for 12 h. After completion, the reaction mixture was diluted with ethyl acetate (20 mL) and washed with saturated sodium bicarbonate and brine successively. The organic layer was dried over anhydrous sodium sulfate and concentrated in vacuo. The residue was purified on preparative thin-layer chromatography (PTLC) to afford the desired product **3**.

## Supporting Information

File 1 General information, experimental procedures for all the products, characterization data, and NMR spectra.

## Data Availability

All data that supports the findings of this study is available in the published article and/or the supporting information to this article.

## References

[1] Michael J P (2008). Nat Prod Rep.

[2] Solomon V R, Lee H (2011). Curr Med Chem.

[3] Bond J A, Walker T W (2012). Weed Technol.

[4] Mohamad N S, Zakaria N H, Daud N, Tan L L, Ta G C, Heng L Y, Hassan N I (2021). Sensors.

[5] Zaitsev V G, Shabashov D, Daugulis O (2005). J Am Chem Soc.

[6] Rouquet G, Chatani N (2013). Angew Chem, Int Ed.

[7] Kuang W-B, Huang R-Z, Fang Y-L, Liang G-B, Yang C-H, Ma X-L, Zhang Y (2018). RSC Adv.

[8] Xu Z, Yang X, Yin S-F, Qiu R (2020). Top Curr Chem.

[9] Zhu L, Cao X, Li Y, Liu T, Wang X, Qiu R, Yin S (2017). Chin J Org Chem.

[10] Suess A M, Ertem M Z, Cramer C J, Stahl S S (2013). J Am Chem Soc.

[11] Long Y, Pan L, Zhou X (2019). Molecules.

[12] He X, Xu Y-z, Kong L-x, Wu H-h, Ji D-z, Wang Z-b, Xu Y-g, Zhu Q-h (2017). Org Chem Front.

[13] Zhu L, Qiu R, Cao X, Xiao S, Xu X, Au C-T, Yin S-F (2015). Org Lett.

[14] Rao N S, Reddy G M, Sridhar B, Sarma M H (2017). Eur J Org Chem.

[15] Chen X-X, Wang J-X, Ren J-T, Xie H, Zhao Y, Li Y-M, Sun M (2017). Synlett.

[16] Jiao J-Y, Mao Y-J, Feng A-W, Li X-F, Li M-T, Zhang X-H (2017). Tetrahedron.

[17] Qiao H, Sun S, Yang F, Zhu Y, Kang J, Wu Y, Wu Y (2017). Adv Synth Catal.

[18] Qiao L, Cao X, Chai K, Shen J, Xu J, Zhang P (2018). Tetrahedron Lett.

[19] Yang X, Yang Q-L, Wang X-Y, Xu H-H, Mei T-S, Huang Y, Fang P (2020). J Org Chem.

[20] Zhao H-Y, Yang X-Y, Lei H, Xin M, Zhang S-Q (2019). Synth Commun.

[21] Li Y, Zhu L, Cao X, Au C-T, Qiu R, Yin S-F (2017). Adv Synth Catal.

[22] Dutta H S, Khan B, Khan A A, Raziullah, Ahmad A, Kant R, Koley D (2017). ChemistrySelect.

[23] Motati D R, Uredi D, Watkins E B (2018). Chem Sci.

[24] He X, Jiang Y, Zhou H, Zhu Q (2021). ChemistrySelect.

[25] Du Y, Liu Y, Wan J-P (2018). J Org Chem.

[26] Li D, Jia Z, Jiang Y, Jia J, Zhao X, Li Z, Xu Z (2019). ChemistrySelect.

[27] Ma B, Lu F, Yang H, Gu X, Li Z, Li R, Pei H, Luo D, Zhang H, Lei A (2019). Asian J Org Chem.

[28] Lin X, Zeng C, Liu C, Fang Z, Guo K (2021). Org Biomol Chem.

[29] Huang B, Zhao Y, Yang C, Gao Y, Xia W (2017). Org Lett.

[30] Shao C, Xu T, Chen C, Yang Q, Tang C, Chen P, Lu M, Hu Z, Hu H, Zhang T (2023). RSC Adv.

[31] Li J-Q, Tan H-L, Ma D-D, Zhu X-X, Cui H-L (2021). J Org Chem.

[32] Liang Y-F, Wu K, Song S, Li X, Huang X, Jiao N (2015). Org Lett.

