# Supplementary material for: Copper-promoted C5-selective bromination of 8-aminoquinoline amides with alkyl bromides
Source: Beilstein J Org Chem. 2024 Jan 23;20:155–61. doi: 10.3762/bjoc.20.14 (PMC10825800; doi:10.3762/bjoc.20.14)
Supplement: File 1 — General information, experimental procedures for all the products, characterization data, and NMR spectra. [file Beilstein_J_Org_Chem-20-155-s001.pdf]

BEILSTEIN JOURNAL OF ORGANIC CHEMISTRY

Supporting Information

for

Copper-promoted C5-selective bromination of 8-aminoquinoline amides with alkyl bromides

Changdong Shao, Chen Ma, Li Li, Jingyi Liu, Yanan Shen, Chen Chen, Qionglin Yang, Tianyi Xu, Zhengsong Hu, Yuhe Kan and Tingting Zhang

Beilstein J. Org. Chem. **2024,** *20,* 155–161. doi:10.3762/bjoc.20.14

General information, experimental procedures for all the products, characterization data, and NMR spectra

License and Terms: This is a supporting information file under the terms of the Creative Commons Attribution License (https://creativecommons.org/licenses/by/4.0). Please note that the reuse, redistribution and reproduction in particular requires that the author(s) and source are credited and that individual graphics may be subject to special legal provisions.
The license is subject to the *Beilstein Journal of Organic Chemistry* terms and conditions: (https://www.beilstein-journals.org/bjoc/terms)

Table of contents

1. General information S2
2. General procedure for the copper-promoted C5-bromination S2
3. Characterization of isolated products S2
4. Copies of NMR spectra S11

5. References S35

1. General information

^{1}H NMR and ^{13}C NMR spectra were recorded on a Bruker ARX400 instrument. NMR spectra were recorded in $CDCl_3$. ^{1}H NMR spectra were referenced to residual $CHCl_3$ at 7.26 ppm, and ^{13}C NMR spectra were referenced to the central peak of $CDCl_3$ at 77.16 ppm. Chemical shifts (δ) are reported in ppm, and coupling constants (J) are in Hertz (Hz). Multiplicities are reported using the following abbreviations: s = singlet, d = doublet, t = triplet, q = quartet, m = multiplet. High resolution mass spectra were obtained on a Bruker micrOTOF II ESI mass spectrometer.

8-Aminoquinoline amides were synthesized according to the previously reported protocol. [1-2] All other chemicals were purchased from commercial sources and used directly without further purification.

2. General procedure for the copper-promoted C5-bromination

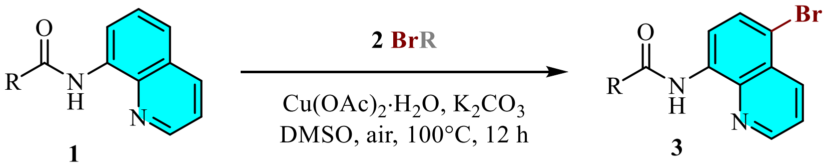

A 35 mL sealed tube equipped with a stirring bar was charged with 8-amidoquinolines (0.2 mmol, 1.0 equiv), BrR (0.8 mmol, 4.0 equiv), $Cu(OAc)_2\cdot H_2O$ (8.0 mg, 0.04 mmol, 20 mol %), K_2CO_3 (27.6 mg, 0.2 mmol, 1.0 equiv), and DMSO (1.0 mL). The tube was sealed with a Teflon cap under air, and the mixture was stirred at 100 °C for 12 h. After completion, the reaction mixture was diluted with ethyl acetate (20 mL) and washed with saturated sodium bicarbonate and brine successively. The organic layer was dried over anhydrous sodium sulfate and concentrated in vacuo. The residue was purified by preparative thin layer chromatography (PTLC) to afford the desired products **3**.

3. Characterization of isolated products

N-(5-Bromoquinolin-8-yl)benzamide (**3aa**)

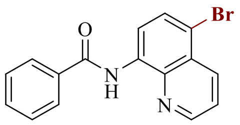

Purified with PTLC (PE/EA = 5/1, R_f = 0.67) to afford the title compound as a white solid (64.5 mg, 99%). **^{1}H NMR (400 MHz, $CDCl_3$)** δ 10.63 (s, 1H), 8.83 – 8.74 (m, 2H), 8.46 (dd, J = 8.5, 1.6 Hz, 1H), 8.08 – 8.01 (m, 2H), 7.78 (d, J = 8.4 Hz, 1H), 7.61 – 7.47 (m, 4H). **^{13}C NMR (101 MHz, $CDCl_3$)** δ 165.33, 148.78, 139.40, 135.97, 134.87, 134.51, 132.06, 130.97, 128.90, 127.33, 127.23, 122.75, 117.01, 114.46. Characterization data were consistent with a previous report. [3]

N-(5-Bromoquinolin-8-yl)-2-methylbenzamide (**3ba**)

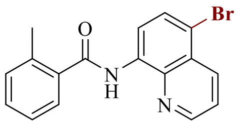

Purified with PTLC (PE/EA = 5/1, R_f = 0.67) to afford the title compound as a white solid (67.6 mg, 99%). **^{1}H NMR (400 MHz, CDCl$_3$)** δ 10.18 (s, 1H), 8.82 (d, *J* = 8.4 Hz, 1H), 8.76 (dd, *J* = 4.2, 1.6 Hz, 1H), 8.49 (dd, *J* = 8.5, 1.6 Hz, 1H), 7.82 (d, *J* = 8.4 Hz, 1H), 7.68 (d, *J* = 7.7 Hz, 1H), 7.52 (dd, *J* = 8.5, 4.2 Hz, 1H), 7.40 (td, *J* = 7.5, 1.5 Hz, 1H), 7.32 (t, *J* = 7.7 Hz, 2H), 2.60 (s, 3H). **^{13}C NMR (101 MHz, CDCl$_3$)** δ 168.15, 148.81, 139.34, 136.88, 136.36, 136.00, 134.72, 131.55, 130.97, 130.58, 127.30, 127.29, 126.13, 122.77, 117.03, 114.55, 20.32. Characterization data were consistent with a previous report. [3]

N-(5-Bromoquinolin-8-yl)-3-methoxybenzamide (**3ca**)

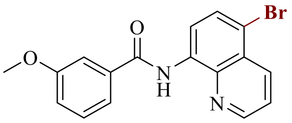

Purified with PTLC (PE/EA = 5/1, R_f = 0.50) to afford the title compound as a white solid (70.5 mg, 99%). **^{1}H NMR (400 MHz, CDCl$_3$)** δ 10.62 (s, 1H), 8.80 (dd, *J* = 4.2, 1.6 Hz, 1H), 8.76 (d, *J* = 8.4 Hz, 1H), 8.46 (dd, *J* = 8.5, 1.6 Hz, 1H), 7.78 (d, *J* = 8.4 Hz, 1H), 7.60 – 7.56 (m, 2H), 7.51 (dd, *J* = 8.5, 4.2 Hz, 1H), 7.42 (t, *J* = 8.2 Hz, 1H), 7.13 – 7.06 (m, 1H), 3.89 (s, 3H). **^{13}C NMR (101 MHz, CDCl$_3$)** δ 165.13, 160.07, 148.79, 139.36, 136.30, 135.93, 134.46, 130.93, 129.85, 127.19, 122.75, 119.05, 118.13, 116.98, 114.47, 112.79, 52.24. Characterization data were consistent with a previous report. [7]

N-(5-Bromoquinolin-8-yl)-4-(trifluoromethyl)benzamide (**3da**)

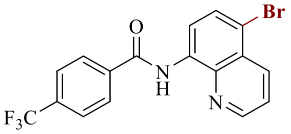

Purified with PTLC (PE/EA = 5/1, R_f = 0.70) to afford the title compound as a white solid (75.0 mg, 95%). **^{1}H NMR (400 MHz, CDCl$_3$)** δ 10.68 (s, 1H), 8.82 (dd, *J* = 4.3, 1.6 Hz, 1H), 8.75 (d, *J* = 8.4 Hz, 1H), 8.50 (dd, *J* = 8.5, 1.6 Hz, 1H), 8.13 (d, *J* = 8.1 Hz, 2H), 7.79 (d, *J* = 8.3 Hz, 3H), 7.56 (dd,

J = 8.5, 4.2 Hz, 1H). **^{13}C NMR (101 MHz, CDCl$_3$)** δ 163.96, 149.00, 139.39, 138.15 (q, J = 1.1 Hz), 136.18, 134.12, 133.74 (q, J = 32.7 Hz), 131.00, 127.84, 127.34, 126.01 (q, J = 3.7 Hz), 123.80 (q, J = 272.7 Hz), 122.95, 117.27, 115.08. Characterization data were consistent with a previous report. [3]

N-(5-Bromoquinolin-8-yl)-4-(*tert*-butyl)benzamide (**3ea**)

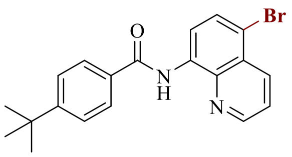

Purified with PTLC (PE/EA = 5/1, R_f = 0.70) to afford the title compound as a white solid (75.0 mg, 98%). **^{1}H NMR (400 MHz, CDCl$_3$)** δ 10.63 (s, 1H), 8.82 – 8.75 (m, 2H), 8.46 (dd, J = 8.5, 1.6 Hz, 1H), 8.03 – 7.96 (m, 2H), 7.78 (d, J = 8.4 Hz, 1H), 7.58 – 7.53 (m, 2H), 7.51 (dd, J = 8.5, 4.2 Hz, 1H), 1.37 (s, 9H). **^{13}C NMR (101 MHz, CDCl$_3$)** δ 165.42, 155.67, 148.76, 139.47, 136.00, 134.70, 132.12, 131.03, 127.27, 127.24, 125.88, 122.75, 116.99, 114.28, 35.13, 31.28. Characterization data were consistent with a previous report. [4]

N-(5-Bromoquinolin-8-yl)-3-fluorobenzamide (**3fa**)

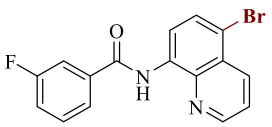

Purified with PTLC (PE/EA = 5/1, R_f = 0.62) to afford the title compound as a white solid (63.5 mg, 92%). **^{1}H NMR (400 MHz, CDCl$_3$)** δ 10.62 (s, 1H), 8.82 (dd, J = 4.2, 1.6 Hz, 1H), 8.74 (d, J = 8.4 Hz, 1H), 8.48 (dd, J = 8.5, 1.6 Hz, 1H), 7.83 – 7.76 (m, 2H), 7.76 – 7.71 (m, 1H), 7.55 (dd, J = 8.5, 4.2 Hz, 1H), 7.52 – 7.45 (m, 1H), 7.33 – 7.20 (m, 1H). **^{13}C NMR (101 MHz, CDCl$_3$)** δ 163.98 (d, J = 2.6 Hz), 163.06 (d, J = 248.0 Hz), 148.95, 139.41, 137.18 (d, J = 6.7 Hz), 136.12, 134.25, 131.00, 130.61 (d, J = 7.8 Hz), 127.32, 122.90, 122.79 (d, J = 3.2 Hz), 119.13 (d, J = 21.3 Hz), 117.19, 114.85, 114.79 (d, J = 23.1 Hz). Characterization data were consistent with a previous report. [3]

N-(5-Bromoquinolin-8-yl)-3-chlorobenzamide (**3ga**)

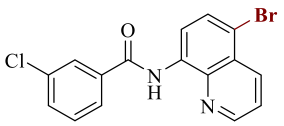

Purified with PTLC (PE/EA = 5/1, R_f = 0.62) to afford the title compound as a white solid (71.6 mg, 99%). **^{1}H NMR (400 MHz, CDCl$_3$)** δ 10.63 (s, 1H), 8.86 (dd, *J* = 4.3, 1.6 Hz, 1H), 8.77 (d, *J* = 8.4 Hz, 1H), 8.53 (dd, *J* = 8.5, 1.6 Hz, 1H), 8.03 (t, *J* = 1.9 Hz, 1H), 7.91 (d, *J* = 7.7 Hz, 1H), 7.82 (d, *J* = 8.4 Hz, 1H), 7.62 – 7.52 (m, 2H), 7.48 (t, *J* = 7.8 Hz, 1H). **^{13}C NMR (101 MHz, CDCl$_3$)** δ 164.03, 149.02, 139.48, 136.75, 136.20, 135.23, 134.29, 132.17, 131.05, 130.25, 127.83, 127.39, 125.37, 122.95, 117.29, 114.94. Characterization data were consistent with a previous report. [3]

4-Bromo-*N*-(5-bromoquinolin-8-yl)benzamide (**3ha**)

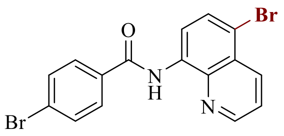

Purified with PTLC (PE/EA = 5/1, R_f = 0.65) to afford the title compound as a white solid (80.4 mg, 99%). **^{1}H NMR (400 MHz, CDCl$_3$)** δ 10.63 (s, 1H), 8.84 (dd, *J* = 4.2, 1.6 Hz, 1H), 8.76 (d, *J* = 8.4 Hz, 1H), 8.52 (dd, *J* = 8.5, 1.6 Hz, 1H), 7.95 – 7.87 (m, 2H), 7.81 (d, *J* = 8.3 Hz, 1H), 7.70 – 7.62 (m, 2H), 7.57 (dd, *J* = 8.5, 4.2 Hz, 1H). **^{13}C NMR (101 MHz, CDCl$_3$)** δ 164.42, 148.94, 139.46, 136.19, 134.34, 133.79, 132.21, 131.07, 128.97, 127.37, 126.96, 122.91, 117.20, 114.81. Characterization data were consistent with a previous report. [3]

N-(5-Bromoquinolin-8-yl)-4-iodobenzamide (**3ia**)

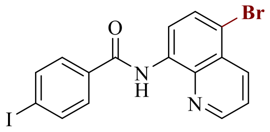

Purified with PTLC (PE/EA = 5/1, R_f = 0.67) to afford the title compound as a white solid (89.5 mg, 99%). **^{1}H NMR (400 MHz, CDCl$_3$)** δ 10.64 (s, 1H), 8.84 (d, *J* = 3.5 Hz, 1H), 8.77 (d, *J* = 8.4 Hz, 1H), 8.52 (d, *J* = 8.1 Hz, 1H), 7.88 (d, *J* = 8.3 Hz, 2H), 7.82 (d, *J* = 8.4 Hz, 1H), 7.76 (d, *J* = 8.4 Hz, 2H), 7.57 (dd, *J* = 8.5, 4.2 Hz, 1H). **^{13}C NMR (101 MHz, CDCl$_3$)** δ 164.64, 148.95, 139.47, 138.21, 136.19, 134.38, 134.34, 131.07, 128.93, 127.38, 122.92, 117.21, 114.82, 99.30. Characterization data were consistent with a previous report. [8]

3-((5-Bromoquinolin-8-yl)carbamoyl)phenyl acetate (**3ja**)

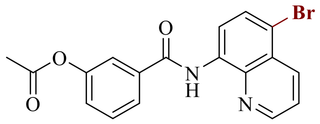

Purified with PTLC (PE/EA = 5/1, R_f = 0.34) to afford the title compound as a white solid (71.0 mg, 92%). **^{1}H NMR (400 MHz, CDCl$_3$)** δ 10.66 (s, 1H), 8.86 (dd, *J* = 4.2, 1.6 Hz, 1H), 8.80 (d, *J* = 8.4 Hz, 1H), 8.54 (dd, *J* = 8.5, 1.6 Hz, 1H), 7.91 (ddd, *J* = 7.8, 1.7, 1.0 Hz, 1H), 7.84 (d, *J* = 8.4 Hz, 1H), 7.78 (t, *J* = 2.0 Hz, 1H), 7.62 – 7.52 (m, 2H), 7.34 (ddd, *J* = 8.1, 2.4, 1.0 Hz, 1H), 2.36 (s, 3H). **^{13}C NMR (101 MHz, CDCl$_3$)** δ 169.34, 164.42, 151.23, 148.98, 139.53, 136.64, 136.18, 134.43, 131.07, 129.99, 127.40, 125.53, 124.51, 122.91, 121.06, 117.28, 114.79, 21.26. Characterization data were consistent with a previous report. [8]

Methyl 3-((5-bromoquinolin-8-yl)carbamoyl)benzoate (**3ka**)

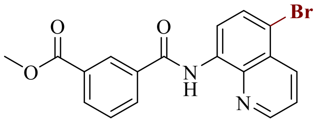

Purified with PTLC (PE/EA = 5/1, R_f = 0.34) to afford the title compound as a white solid (71.5 mg, 93%). **^{1}H NMR (400 MHz, CDCl$_3$)** δ 10.68 (s, 1H), 8.85 (dd, *J* = 4.2, 1.6 Hz, 1H), 8.78 (d, *J* = 8.4 Hz, 1H), 8.68 (t, *J* = 1.8 Hz, 1H), 8.50 (dd, *J* = 8.5, 1.6 Hz, 1H), 8.23 (dd, *J* = 7.9, 1.2 Hz, 2H), 7.81 (d, *J* = 8.4 Hz, 1H), 7.61 (t, *J* = 7.8 Hz, 1H), 7.56 (dd, *J* = 8.5, 4.2 Hz, 1H), 3.98 (s, 3H). **^{13}C NMR (101 MHz, CDCl$_3$)** δ 166.37, 164.43, 148.99, 139.45, 136.10, 135.35, 134.34, 132.95, 131.73, 131.02, 131.00, 129.16, 128.38, 127.32, 122.89, 117.27, 114.84, 52.54. Characterization data were consistent with a previous report. [8]

N-(5-Bromoquinolin-8-yl)-2,4,6-trimethylbenzamide (**3la**)

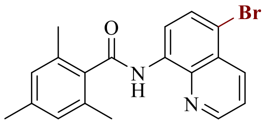

Purified with PTLC (PE/EA = 5/1, R_f = 0.76) to afford the title compound as a white solid (51.5 mg, 70%). **^{1}H NMR (400 MHz, CDCl$_3$)** δ 9.92 (s, 1H), 8.90 (d, *J* = 8.4 Hz, 1H), 8.73 (dd, *J* = 4.2, 1.6 Hz, 1H), 8.51 (dd, *J* = 8.5, 1.6 Hz, 1H), 7.85 (d, *J* = 8.4 Hz, 1H), 7.52 (dd, *J* = 8.5, 4.2 Hz, 1H), 6.92 (s, 2H), 2.40 (s, 6H), 2.33 (s, 3H). **^{13}C NMR (101 MHz, CDCl$_3$)** δ 169.18, 148.79, 139.26, 138.98, 135.94, 135.17, 134.55, 134.46, 130.94, 128.54, 127.29, 122.75, 117.20, 114.63, 21.23, 19.49. Characterization data were consistent with a previous report. [4]

N-(5-Bromoquinolin-8-yl)-3,4,5-trimethoxybenzamide (**3ma**)

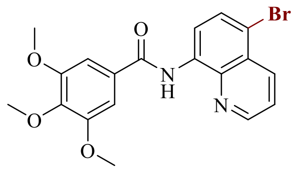

Purified with PTLC (PE/EA = 5/1, R_f = 0.35) to afford the title compound as a white solid (82.5 mg, 99%). **^{1}H NMR (400 MHz, CDCl$_3$)** δ 10.59 (s, 1H), 8.84 (dd, *J* = 4.3, 1.6 Hz, 1H), 8.77 (d, *J* = 8.4 Hz, 1H), 8.54 (dd, *J* = 8.5, 1.6 Hz, 1H), 7.84 (d, *J* = 8.4 Hz, 1H), 7.58 (dd, *J* = 8.5, 4.2 Hz, 1H), 7.27 (s, 2H), 3.99 (s, 6H), 3.93 (s, 3H). **^{13}C NMR (101 MHz, CDCl$_3$)** δ 165.26, 153.53, 148.95, 141.73, 139.54, 136.19, 134.61, 131.12, 130.45, 127.40, 122.86, 117.08, 114.53, 105.01, 61.10, 56.59. Characterization data were consistent with a previous report. [8]

N-(5-Bromoquinolin-8-yl)-1-naphthamide (**3na**)

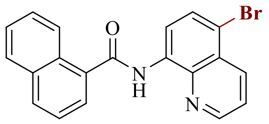

Purified with PTLC (PE/EA = 5/1, R_f = 0.64) to afford the title compound as a white solid (71.5 mg, 95%). **^{1}H NMR (400 MHz, CDCl$_3$)** δ 10.40 (s, 1H), 8.95 (d, *J* = 8.3 Hz, 1H), 8.76 (dd, *J* = 4.2, 1.6 Hz, 1H), 8.58 – 8.49 (m, 2H), 8.02 (d, *J* = 8.3 Hz, 1H), 7.96 – 7.87 (m, 3H), 7.64 – 7.51 (m, 4H). **^{13}C NMR (101 MHz, CDCl$_3$)** δ 166.23, 151.03, 140.13, 136.13, 134.89, 134.50, 134.05, 131.47, 131.10, 130.46, 128.57, 127.54, 127.44, 126.72, 125.70, 125.62, 124.98, 122.87, 117.78, 114.82. Characterization data were consistent with a previous report. [4]

N-(5-Bromoquinolin-8-yl)furan-2-carboxamide (**3oa**)

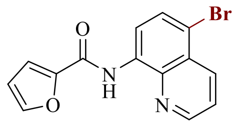

Purified with PTLC (PE/EA = 5/1, R_f = 0.59) to afford the title compound as a white solid (61.0 mg, 96%). **^{1}H NMR (400 MHz, CDCl$_3$)** δ 10.70 (s, 1H), 8.87 (dd, *J* = 4.2, 1.6 Hz, 1H), 8.74 (d, *J* = 8.4 Hz, 1H), 8.51 (dd, *J* = 8.5, 1.6 Hz, 1H), 7.80 (d, *J* = 8.4 Hz, 1H), 7.62 (dd, *J* = 1.8, 0.8 Hz, 1H), 7.56 (dd, *J* = 8.5, 4.2 Hz, 1H), 7.29 (dd, *J* = 3.5, 0.9 Hz, 1H), 6.58 (dd, *J* = 3.5, 1.7 Hz, 1H). **^{13}C NMR (101 MHz, CDCl$_3$)** δ 156.42, 148.99, 148.26, 144.77, 139.40, 136.03, 134.23, 131.00, 127.36, 122.86, 117.19, 115.51, 114.70, 112.65. Characterization data were consistent with a previous report.[5]

N-(5-Bromoquinolin-8-yl)thiophene-2-carboxamide (**3pa**)

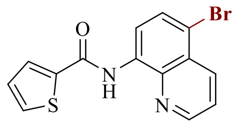

Purified with PTLC (PE/EA = 5/1, R_f = 0.59) to afford the title compound as a white solid (66.0 mg, 99%). **^{1}H NMR (400 MHz, $CDCl_3$)** δ 10.47 (s, 1H), 8.81 (dd, *J* = 4.2, 1.6 Hz, 1H), 8.68 (d, *J* = 8.4 Hz, 1H), 8.47 (dd, *J* = 8.5, 1.6 Hz, 1H), 7.82 – 7.74 (m, 2H), 7.58 (dd, *J* = 5.0, 1.1 Hz, 1H), 7.53 (dd, *J* = 8.5, 4.2 Hz, 1H), 7.16 (dd, *J* = 5.0, 3.7 Hz, 1H). **^{13}C NMR (101 MHz, $CDCl_3$)** δ 159.99, 148.87, 139.84, 139.18, 136.03, 134.29, 131.26, 131.00, 128.68, 128.02, 127.28, 122.83, 117.01, 114.51. Characterization data were consistent with a previous report. [3]

N-(5-Bromoquinolin-8-yl)-2-phenylacetamide (**3qa**)

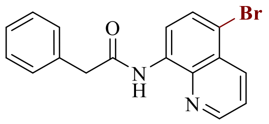

Purified with PTLC (PE/EA = 5/1, R_f = 0.53) to afford the title compound as a white solid (67.5 mg, 99%). **^{1}H NMR (400 MHz, $CDCl_3$)** δ 9.85 (s, 1H), 8.69 – 8.60 (m, 2H), 8.42 (dd, *J* = 8.5, 1.6 Hz, 1H), 7.72 (d, *J* = 8.4 Hz, 1H), 7.49 – 7.38 (m, 5H), 7.38 – 7.32 (m, 1H), 3.88 (s, 2H). **^{13}C NMR (101 MHz, $CDCl_3$)** δ 169.55, 148.67, 139.12, 135.83, 134.55, 134.35, 130.86, 129.63, 129.10, 127.51, 127.10, 122.62, 116.84, 114.34, 45.43. Characterization data were consistent with a previous report. [3]

N-(5-Bromoquinolin-8-yl)acetamide (**3ra**)

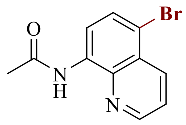

Purified with PTLC (PE/EA = 5/1, R_f = 0.59) to afford the title compound as a white solid (45.5 mg, 86%). **^{1}H NMR (400 MHz, $CDCl_3$)** δ 9.73 (s, 1H), 8.78 (dd, *J* = 4.2, 1.6 Hz, 1H), 8.62 (d, *J* = 8.4 Hz, 1H), 8.48 (dd, *J* = 8.5, 1.6 Hz, 1H), 7.75 (d, *J* = 8.4 Hz, 1H), 7.53 (dd, *J* = 8.5, 4.2 Hz, 1H), 2.34 (s, 3H). **^{13}C NMR (101 MHz, $CDCl_3$)** δ 168.86, 148.67, 138.99, 136.06, 134.53, 131.00, 127.22, 122.73, 116.99, 114.21, 25.24. Characterization data were consistent with a previous report. [6]

N-(5-Bromoquinolin-8-yl)isobutyramide (**3sa**)

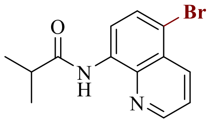

Purified with PTLC (PE/EA = 5/1, R_f = 0.67) to afford the title compound as a white solid (48.0 mg, 82%). **^{1}H NMR (400 MHz, CDCl$_3$)** δ 9.86 (s, 1H), 8.81 (dd, *J* = 4.2, 1.6 Hz, 1H), 8.68 (d, *J* = 8.4 Hz, 1H), 8.50 (dd, *J* = 8.5, 1.6 Hz, 1H), 7.77 (d, *J* = 8.4 Hz, 1H), 7.54 (dd, *J* = 8.5, 4.2 Hz, 1H), 2.76 (hept, *J* = 6.9 Hz, 1H), 1.35 (s, 3H), 1.34 (s, 3H). **^{13}C NMR (101 MHz, CDCl$_3$)** δ 175.84, 148.72, 139.32, 136.07, 134.71, 131.07, 127.29, 122.72, 117.03, 114.06, 37.27, 19.78. Characterization data were consistent with a previous report. [6]

N-(5-Bromoquinolin-8-yl)pivalamide (**3ta**)

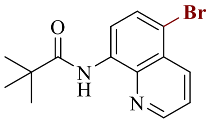

Purified with PTLC (PE/EA = 5/1, R_f = 0.76) to afford the title compound as a white solid (51.0 mg, 83%). **^{1}H NMR (400 MHz, CDCl$_3$)** δ 10.22 (s, 1H), 8.82 (dd, *J* = 4.2, 1.6 Hz, 1H), 8.68 (d, *J* = 8.4 Hz, 1H), 8.50 (dd, *J* = 8.5, 1.6 Hz, 1H), 7.78 (d, *J* = 8.4 Hz, 1H), 7.54 (dd, *J* = 8.5, 4.2 Hz, 1H), 1.42 (s, 9H). **^{13}C NMR (101 MHz, CDCl$_3$)** δ 177.38, 148.81, 139.62, 136.04, 134.79, 131.07, 127.29, 122.69, 116.86, 114.00, 40.51, 27.81. Characterization data were consistent with a previous report. [3]

N-(5-Bromoquinolin-8-yl)cyclopropanecarboxamide (**3ua**)

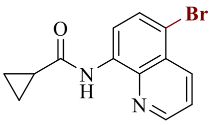

Purified with PTLC (PE/EA = 5/1, R_f = 0.57) to afford the title compound as a white solid (54.0 mg, 93%). **^{1}H NMR (400 MHz, CDCl$_3$)** δ 9.95 (s, 1H), 8.78 (dd, *J* = 4.2, 1.6 Hz, 1H), 8.60 (d, *J* = 8.4 Hz, 1H), 8.47 (dd, *J* = 8.5, 1.6 Hz, 1H), 7.73 (d, *J* = 8.4 Hz, 1H), 7.52 (dd, *J* = 8.5, 4.2 Hz, 1H), 1.82 – 1.74 (m, 1H), 1.19 – 1.07 (m, 2H), 1.00 – 0.82 (m, 2H). **^{13}C NMR (101 MHz, CDCl$_3$)** δ 172.35, 148.61, 138.98, 135.98, 134.72, 131.02, 127.22, 122.68, 116.93, 113.89, 16.40, 8.39. Characterization data were consistent with a previous report. [8]

N-(5-Bromoquinolin-8-yl)cyclobutanecarboxamide (**3va**)

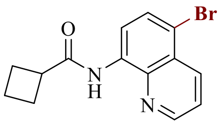

Purified with PTLC (PE/EA = 5/1, R_f = 0.65) to afford the title compound as a white solid (59.8 mg, 98%). **^{1}H NMR (400 MHz, $CDCl_3$)** δ 9.69 (s, 1H), 8.77 (dd, *J* = 4.2, 1.6 Hz, 1H), 8.67 (d, *J* = 8.4 Hz, 1H), 8.47 (dd, *J* = 8.5, 1.6 Hz, 1H), 7.75 (d, *J* = 8.4 Hz, 1H), 7.51 (dd, *J* = 8.5, 4.2 Hz, 1H), 3.37 (p, *J* = 8.5 Hz, 1H), 2.54 – 2.40 (m, 2H), 2.38 – 2.25 (m, 2H), 2.15 – 1.87 (m, 2H). **^{13}C NMR (101 MHz, $CDCl_3$)** δ 173.83, 148.66, 139.18, 136.00, 134.60, 131.02, 127.23, 122.68, 116.93, 114.01, 41.48, 25.57, 18.27. **HRMS (ESI-TOF)** m/z: calculated for $C_{14}H_{13}BrN_2NaO^+$: 327.0103 (M + Na) +, found: 327.0112.

N-(5-Bromoquinolin-8-yl)-4-methylbenzenesulfonamide (**3za**)

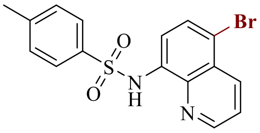

Purified with PTLC (PE/EA = 5/1, R_f = 0.38) to afford the title compound as a white solid (40.0 mg, 53%). **^{1}H NMR (400 MHz, $CDCl_3$)** δ 9.19 (s, 1H), 8.76 (dd, *J* = 4.2, 1.6 Hz, 1H), 8.41 (dd, *J* = 8.5, 1.6 Hz, 1H), 7.78 (d, *J* = 8.4 Hz, 2H), 7.73 – 7.64 (m, 2H), 7.50 (dd, *J* = 8.5, 4.2 Hz, 1H), 7.15 (d, *J* = 8.0 Hz, 2H), 2.29 (s, 3H). **^{13}C NMR (101 MHz, $CDCl_3$)** δ 149.30, 144.11, 139.18, 136.31, 135.98, 133.93, 130.43, 129.73, 127.57, 127.33, 123.13, 115.33, 114.99, 21.57. Characterization data were consistent with a previous report. [9]

N-(5-Bromo-6-methoxyquinolin-8-yl)benzamide (**5**)

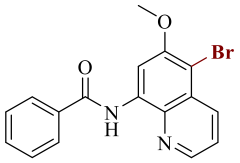

Purified with PTLC (PE/EA = 5/1, R_f = 0.48) to afford the title compound as a white solid (70.5 mg, 99%). **^{1}H NMR (400 MHz, $CDCl_3$)** δ 10.71 (s, 1H), 8.91 (s, 1H), 8.64 (dd, *J* = 4.2, 1.5 Hz, 1H), 8.44 (dd, *J* = 8.6, 1.6 Hz, 1H), 8.09 – 8.01 (m, 2H), 7.63 – 7.50 (m, 3H), 7.46 (dd, *J* = 8.6, 4.2 Hz, 1H), 4.09 (s, 3H). **^{13}C NMR (101 MHz, $CDCl_3$)** δ 165.51, 154.61, 146.31, 135.42, 134.98, 134.77, 134.71, 132.21, 128.98, 128.11, 127.32, 123.17, 104.49, 99.80, 57.08. Characterization data were consistent with a previous report. [3]

4. Copies of NMR spectra

^{1}H NMR for *N*-(5-bromoquinolin-8-yl)benzamide (**3aa**)

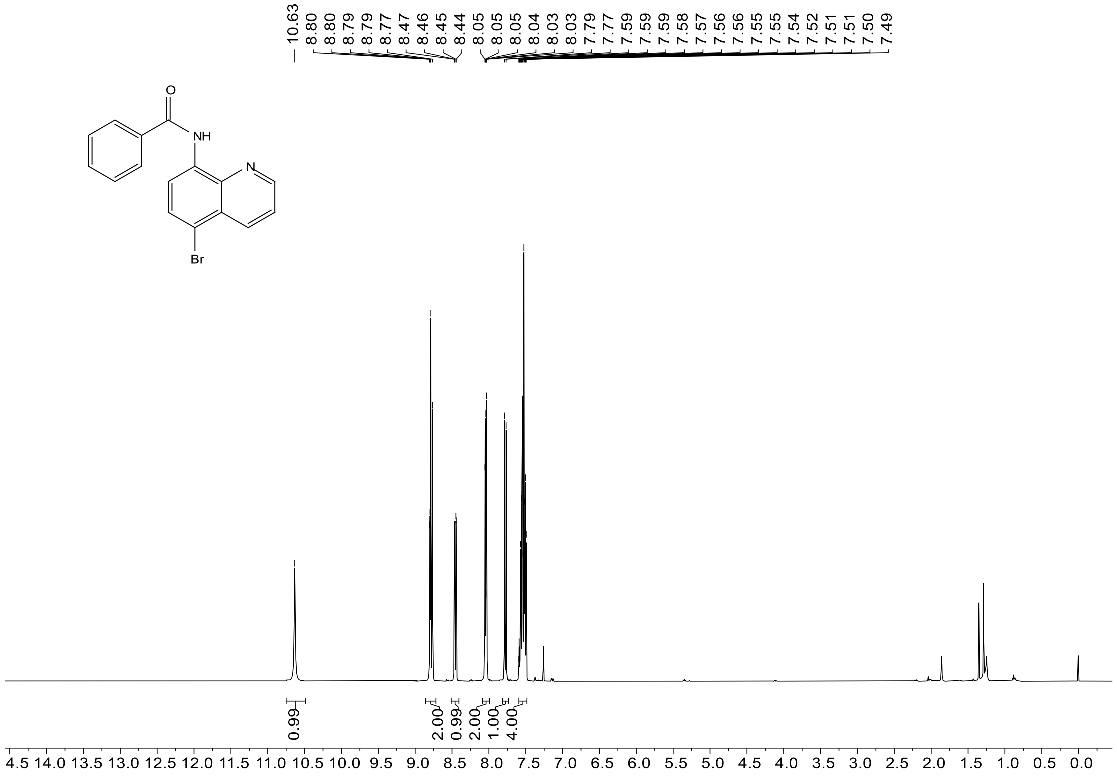

^{13}C NMR for *N*-(5-bromoquinolin-8-yl)benzamide (**3aa**)

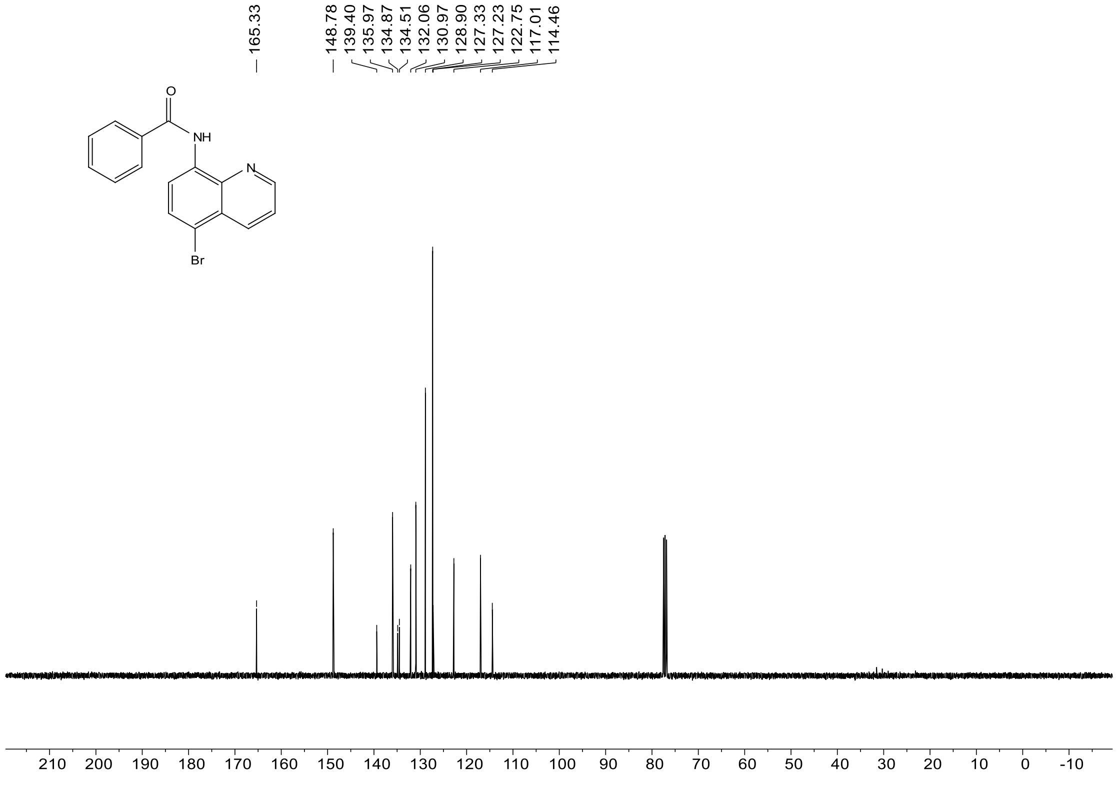

^{1}H NMR for *N*-(5-bromoquinolin-8-yl)-2-methylbenzamide (**3ba**)

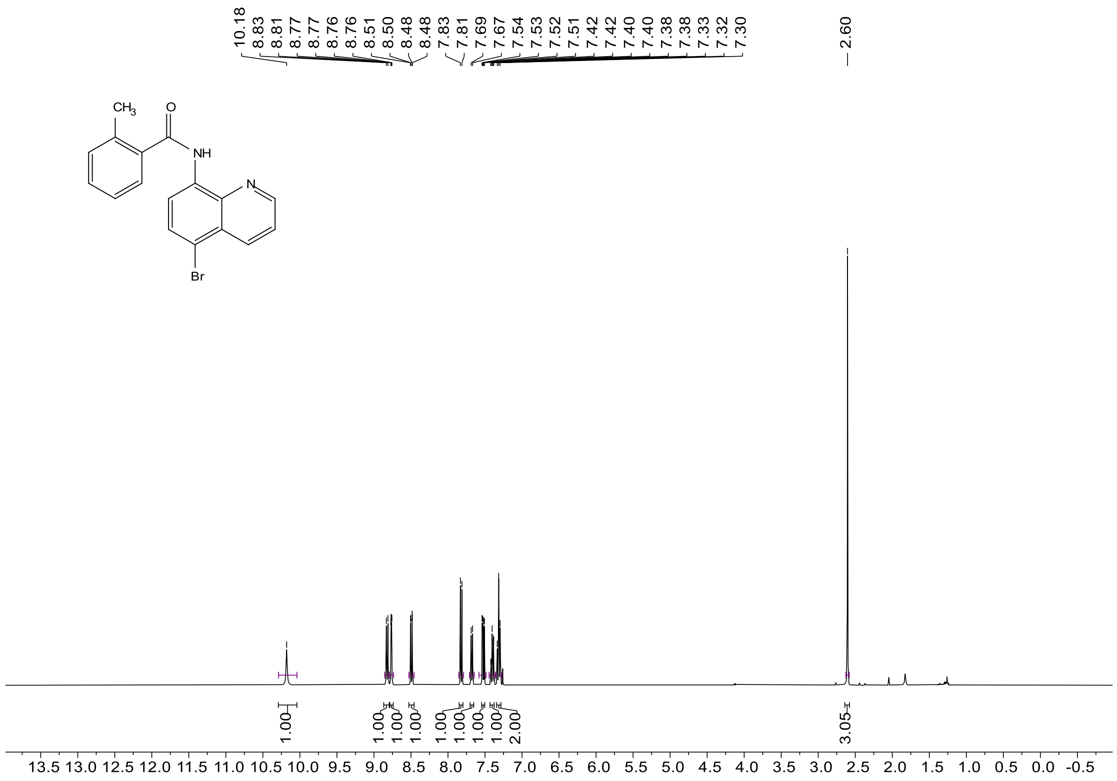

^{13}C NMR for *N*-(5-bromoquinolin-8-yl)-2-methylbenzamide (**3ba**)

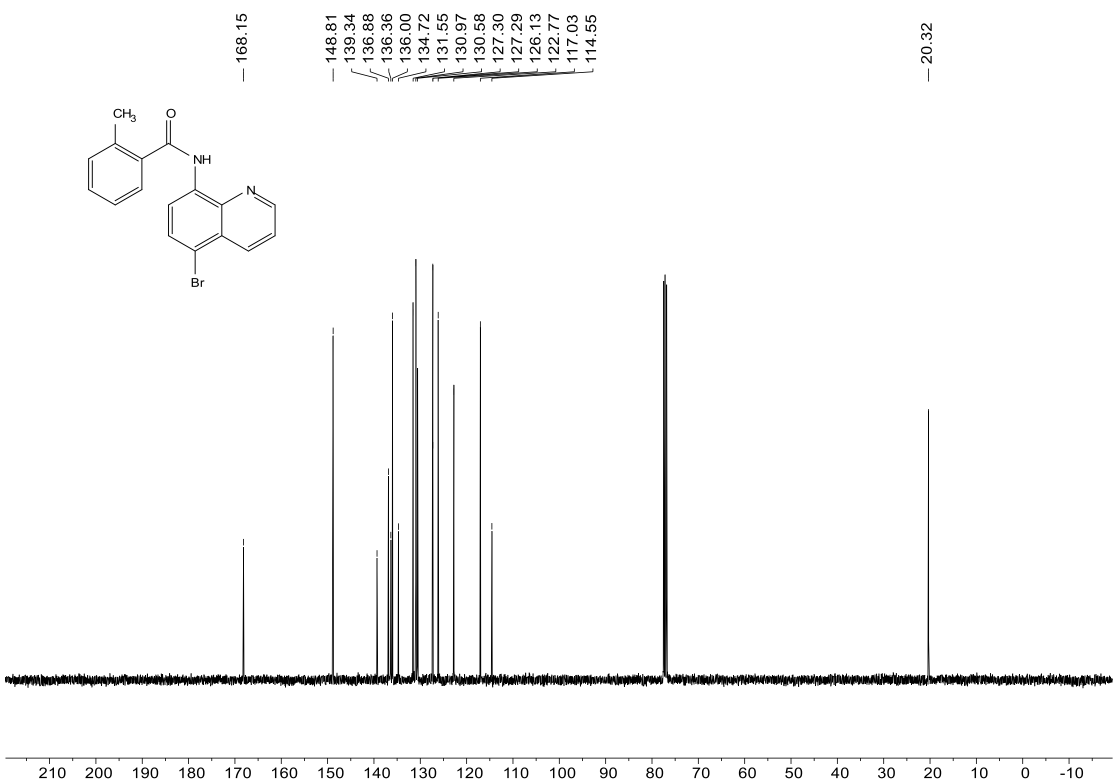

^{1}H NMR for *N*-(5-bromoquinolin-8-yl)-3-methoxybenzamide (**3ca**)

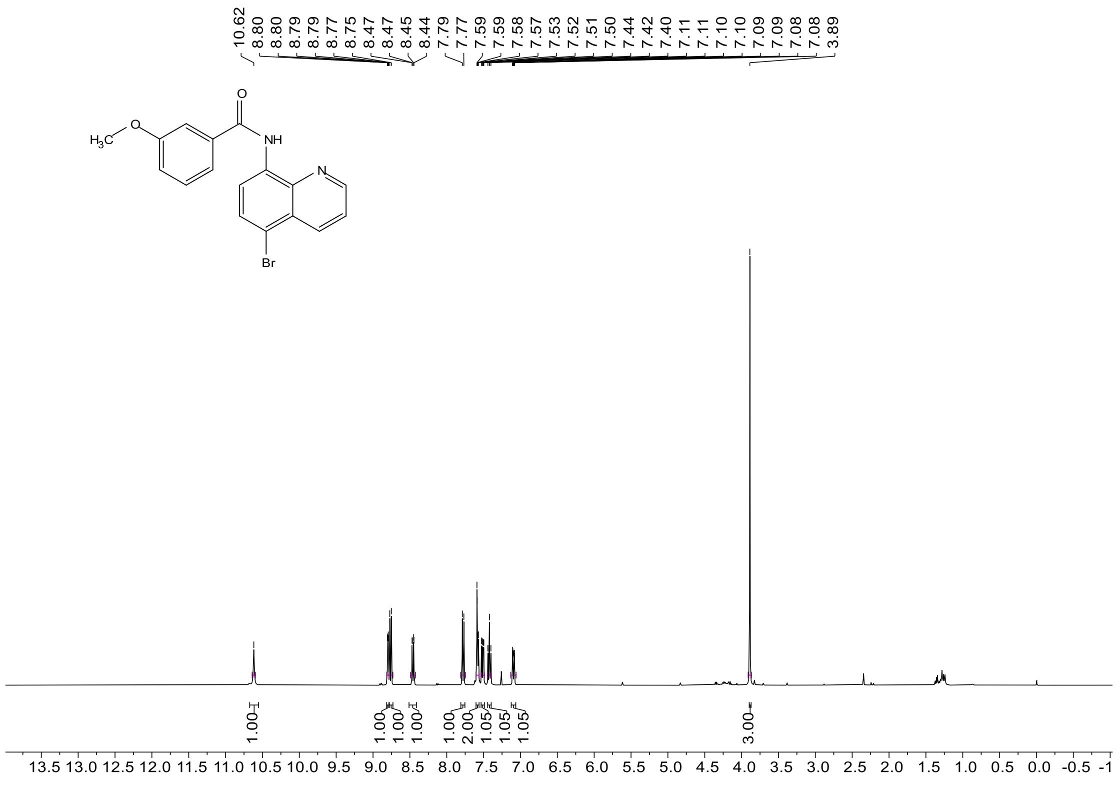

^{13}C NMR for *N*-(5-bromoquinolin-8-yl)-3-methoxybenzamide (**3ca**)

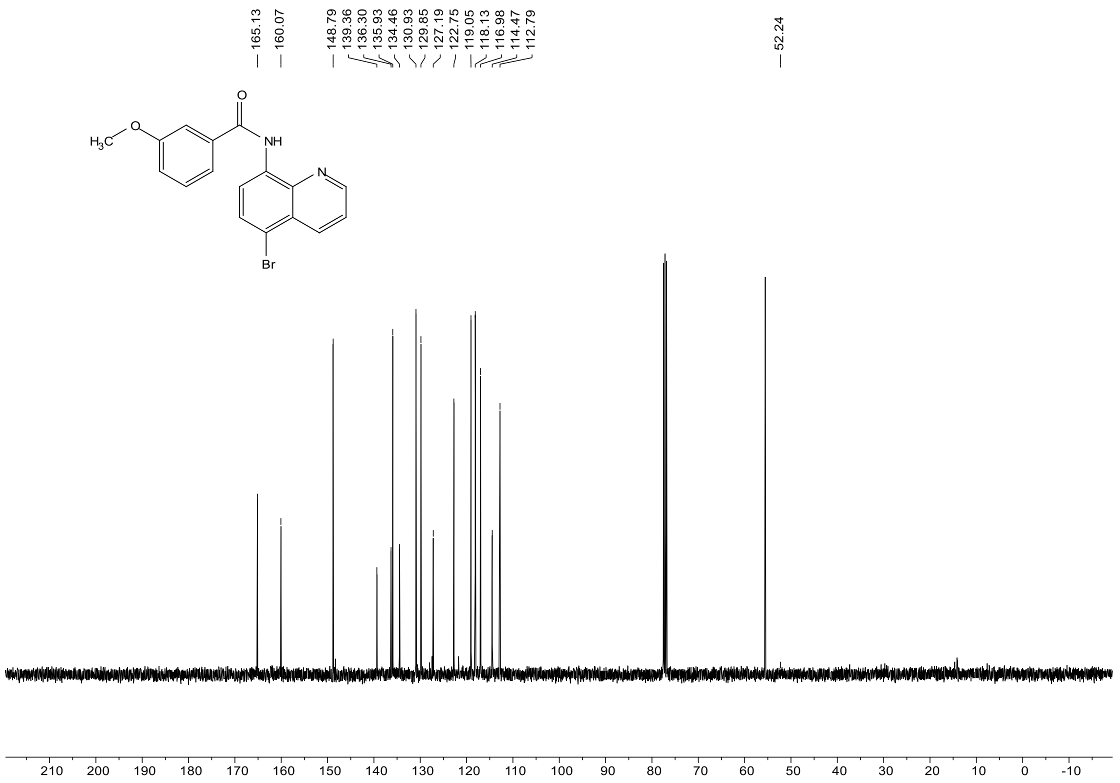

^{1}H NMR for *N*-(5-bromoquinolin-8-yl)-4-(trifluoromethyl)benzamide (**3da**)

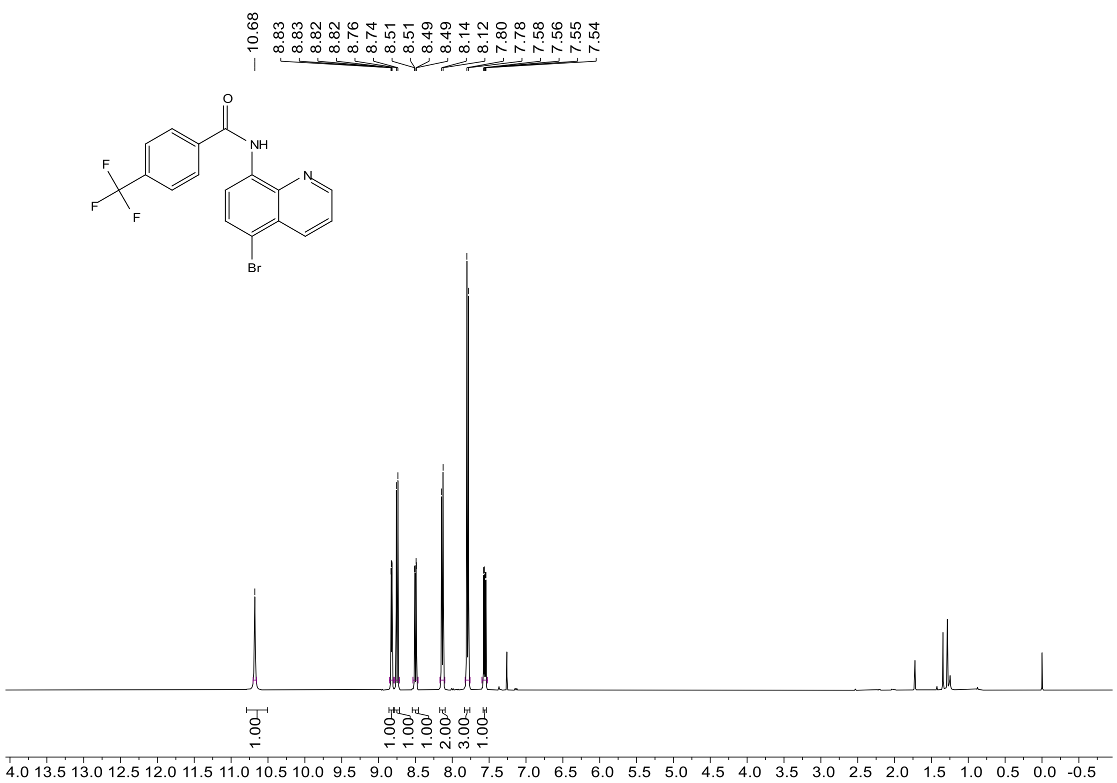

^{13}C NMR for *N*-(5-bromoquinolin-8-yl)-4-(trifluoromethyl)benzamide (**3da**)

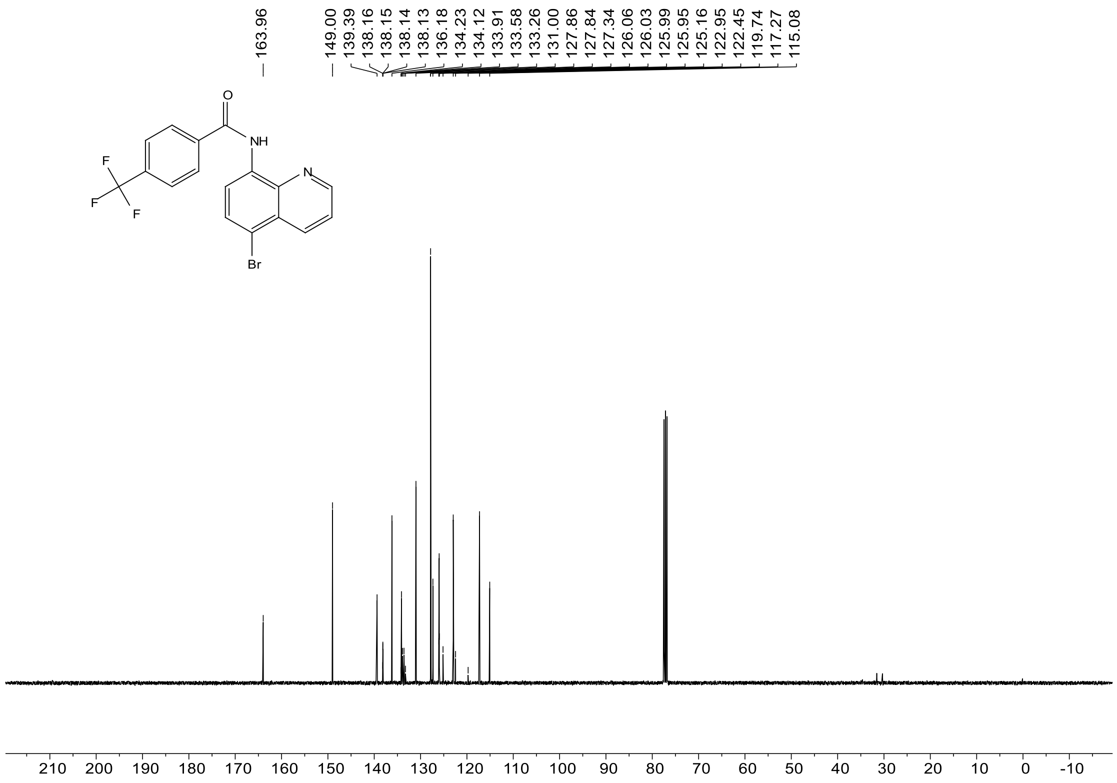

^{1}H NMR for *N*-(5-bromoquinolin-8-yl)-4-(*tert*-butyl)benzamide (**3ea**)

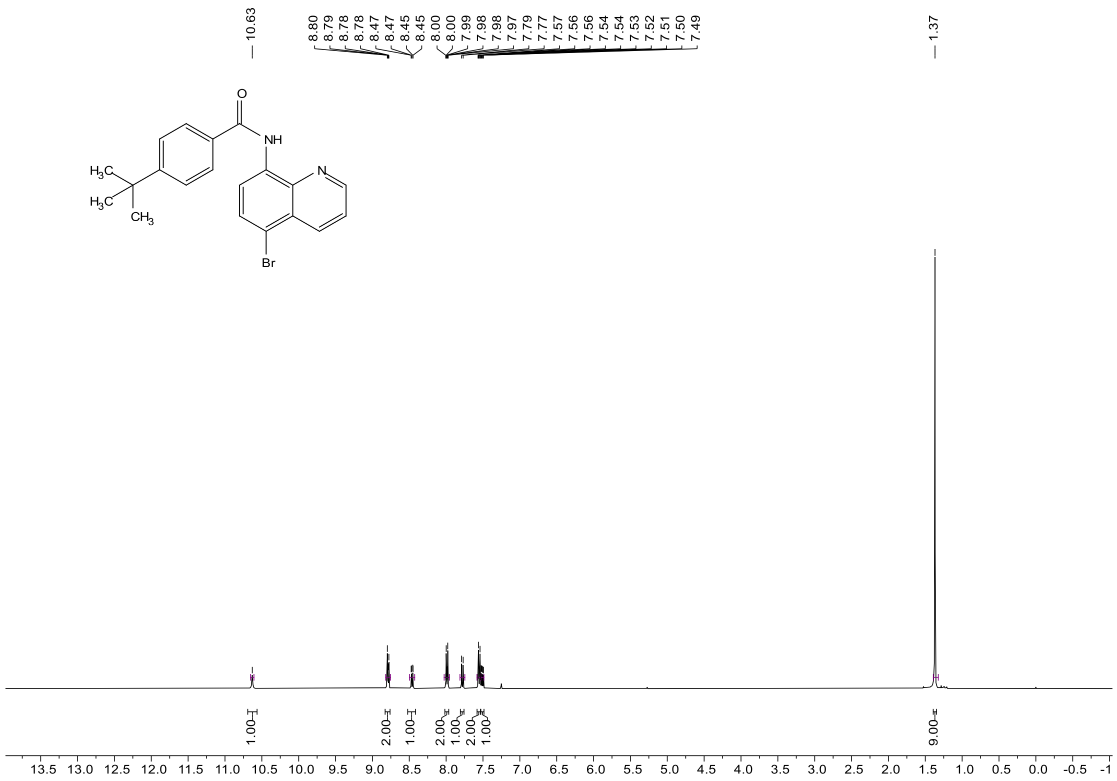

^{13}C NMR for *N*-(5-bromoquinolin-8-yl)-4-(*tert*-butyl)benzamide (**3ea**)

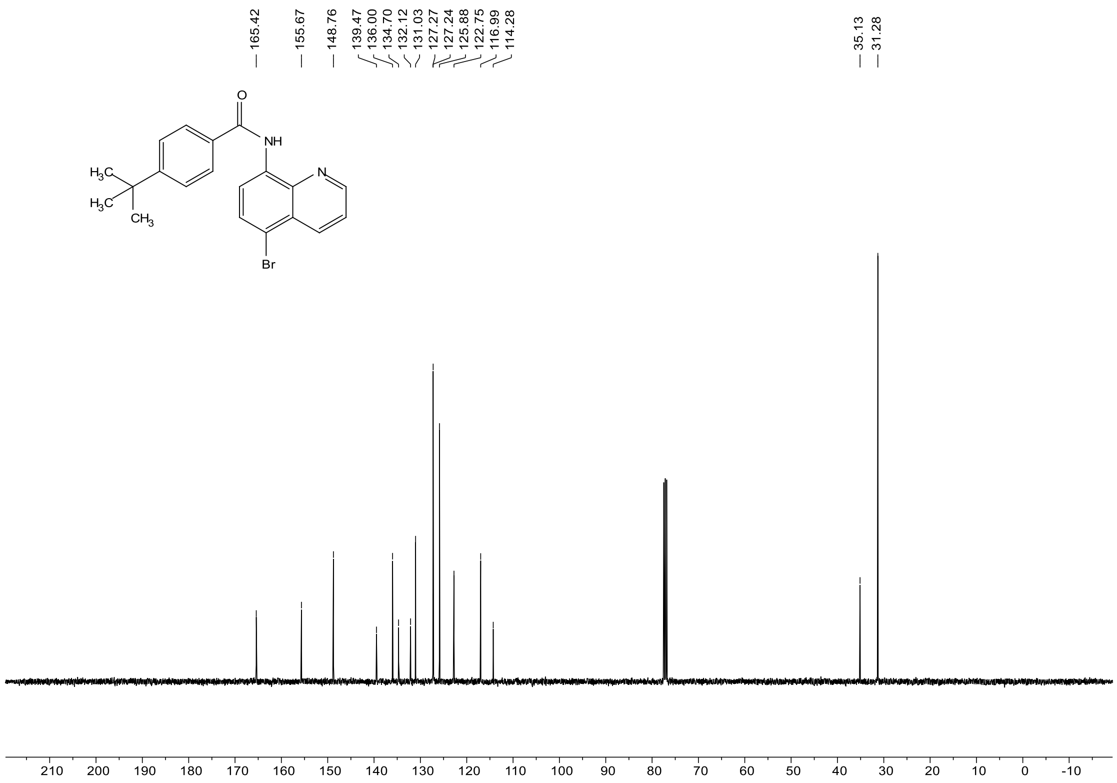

^{1}H NMR for *N*-(5-bromoquinolin-8-yl)-3-fluorobenzamide (**3fa**)

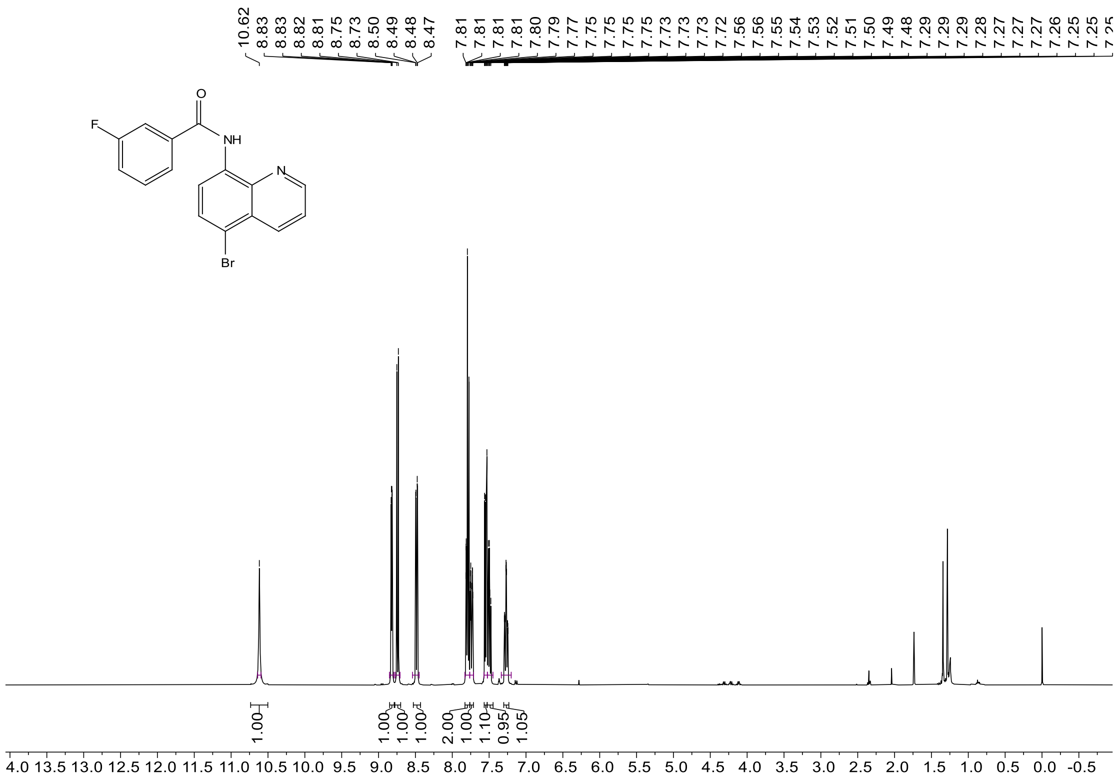

^{13}C NMR for *N*-(5-bromoquinolin-8-yl)-3-fluorobenzamide (**3fa**)

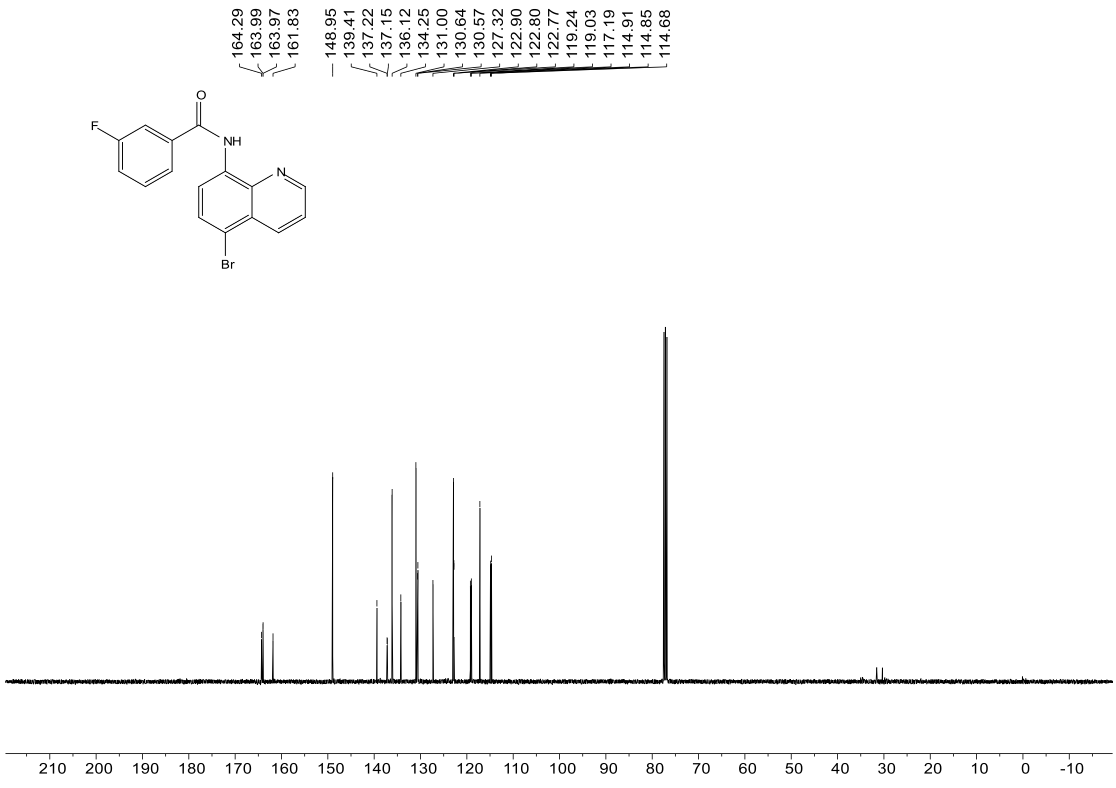

^{1}H NMR for *N*-(5-bromoquinolin-8-yl)-3-chlorobenzamide (**3ga**)

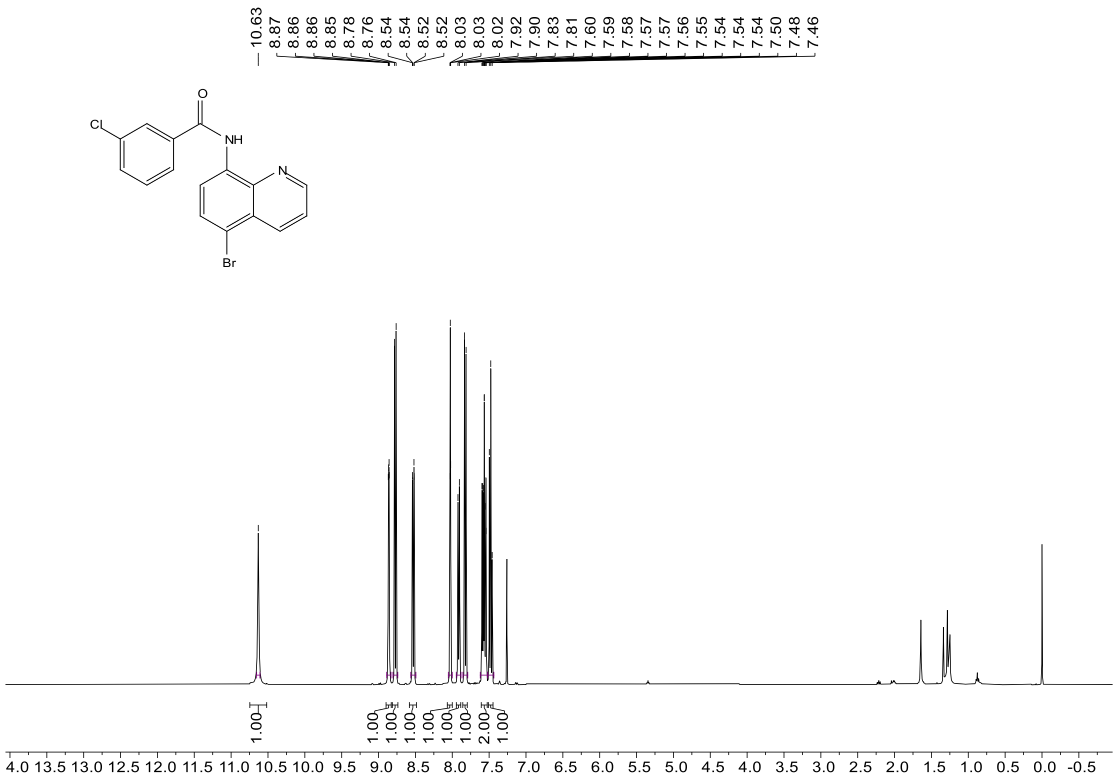

^{13}C NMR for *N*-(5-bromoquinolin-8-yl)-3-chlorobenzamide (**3ga**)

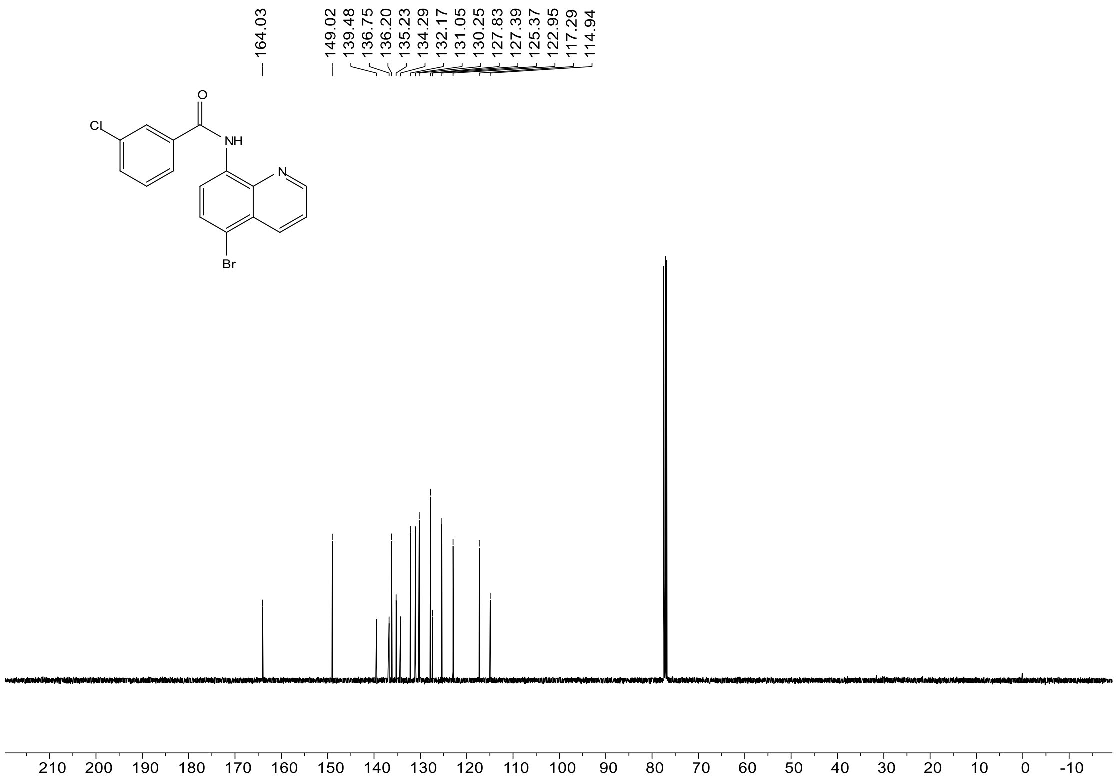

^{1}H NMR for 4-bromo-*N*-(5-bromoquinolin-8-yl)benzamide (**3ha**)

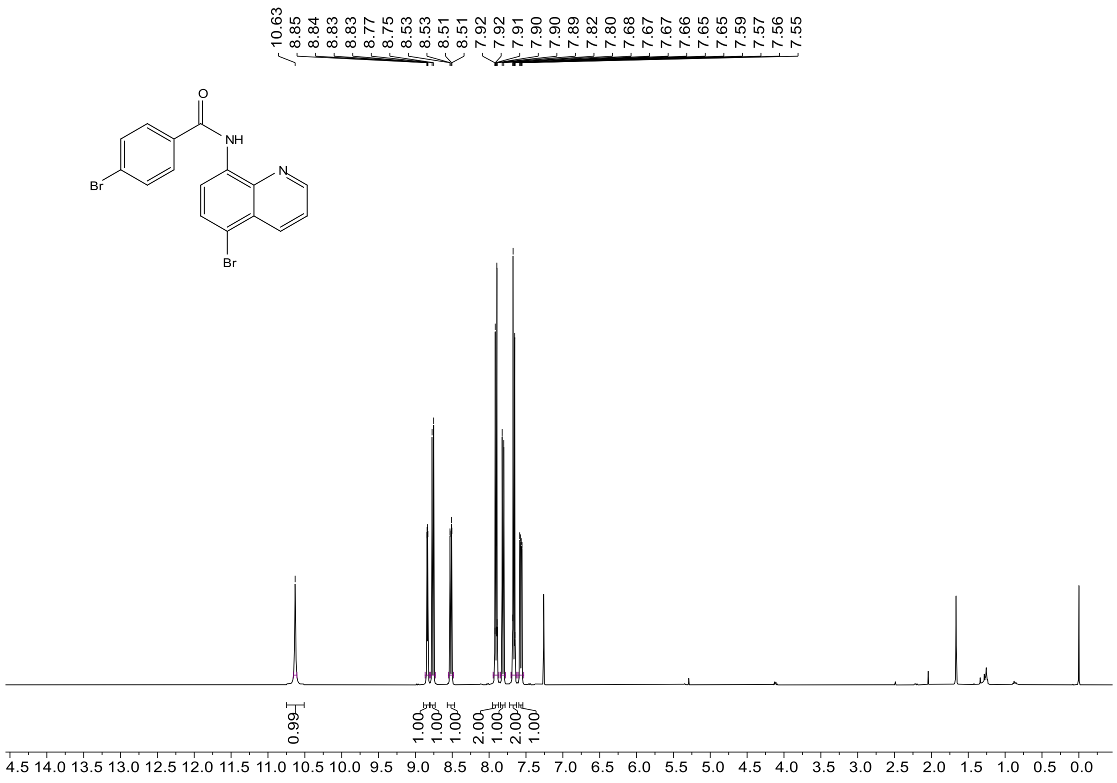

^{13}C NMR for 4-bromo-*N*-(5-bromoquinolin-8-yl)benzamide (**3ha**)

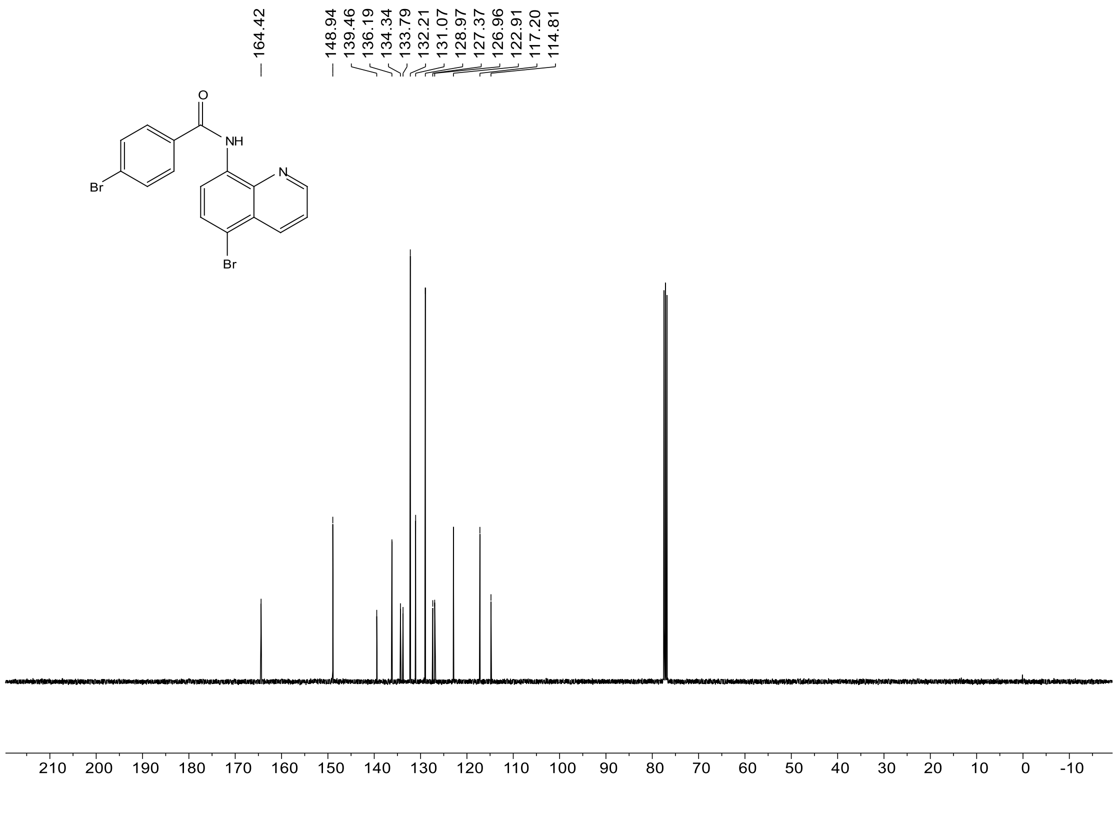

^{1}H NMR for *N*-(5-bromoquinolin-8-yl)-4-iodobenzamide (**3ia**)

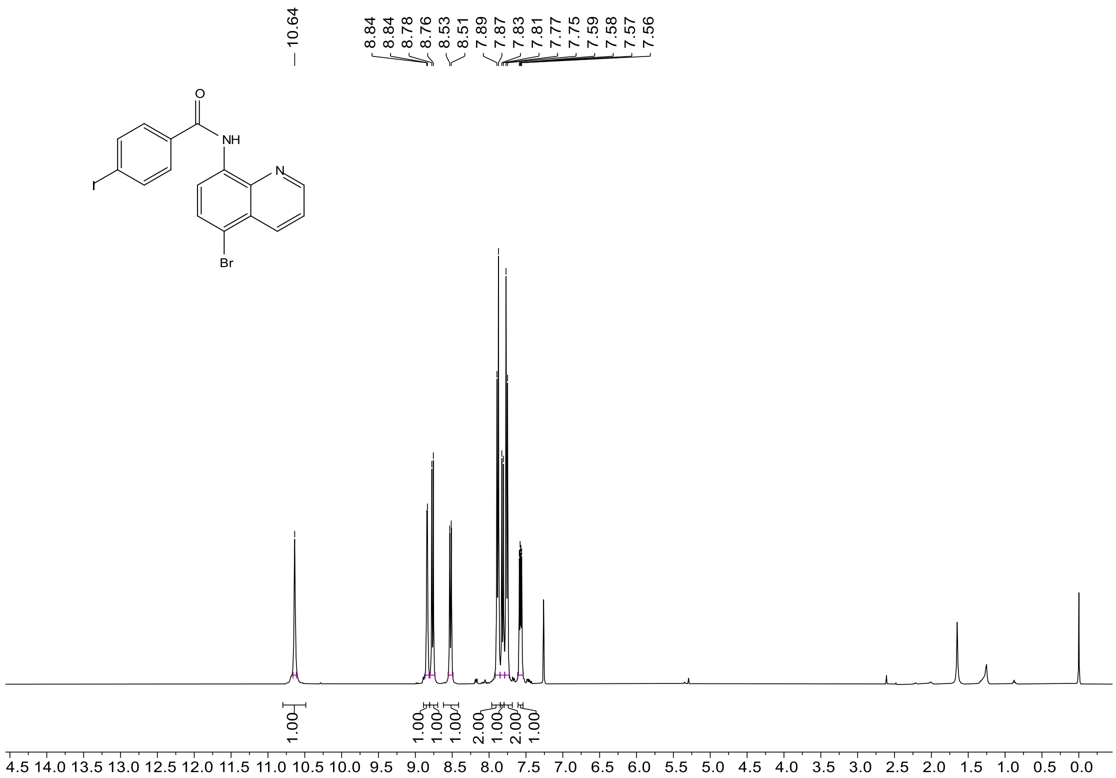

^{13}C NMR for *N*-(5-bromoquinolin-8-yl)-4-iodobenzamide (**3ia**)

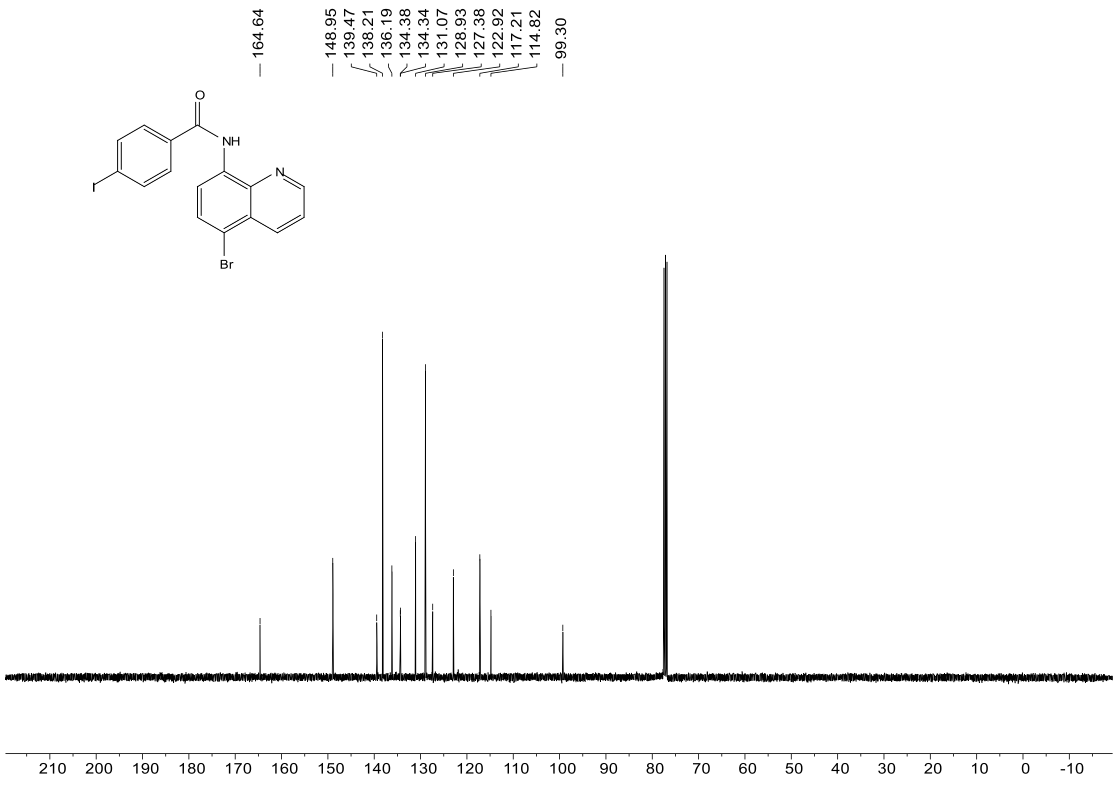

^{1}H NMR for 3-((5-bromoquinolin-8-yl)carbamoyl)phenyl acetate (**3ja**)

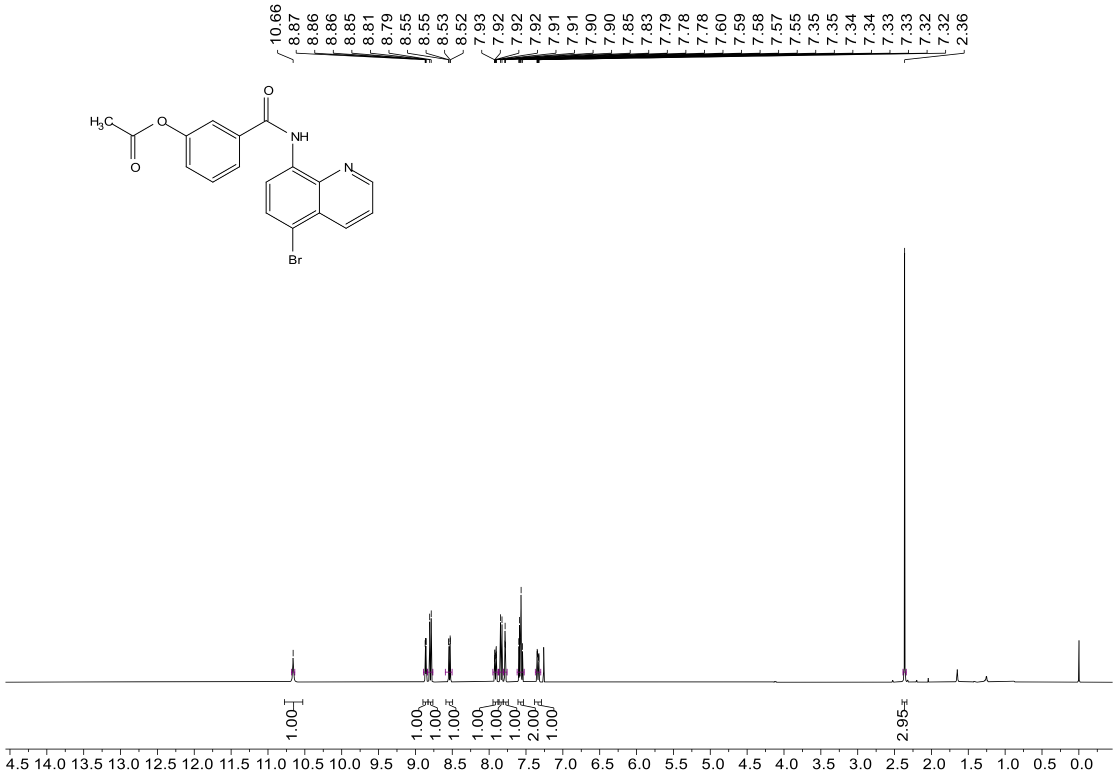

^{13}C NMR for 3-((5-bromoquinolin-8-yl)carbamoyl)phenyl acetate (**3ja**)

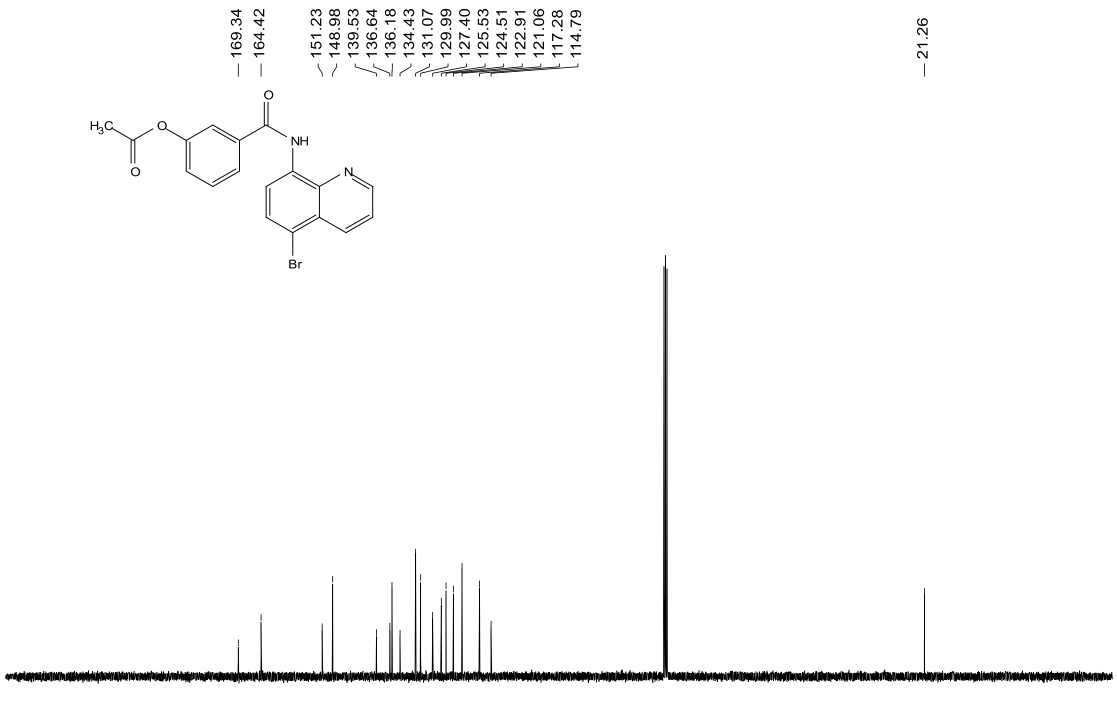

^{1}H NMR for methyl 3-((5-bromoquinolin-8-yl)carbamoyl)benzoate (**3ka**)

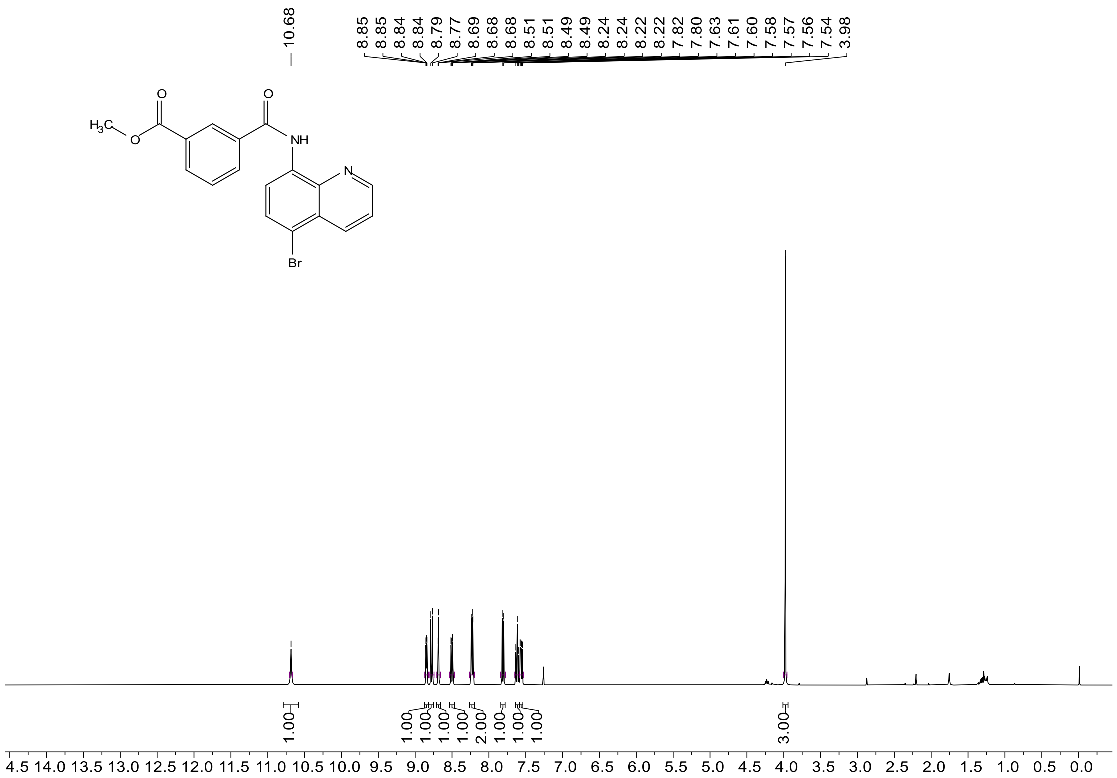

^{13}C NMR for methyl 3-((5-bromoquinolin-8-yl)carbamoyl)benzoate (**3ka**)

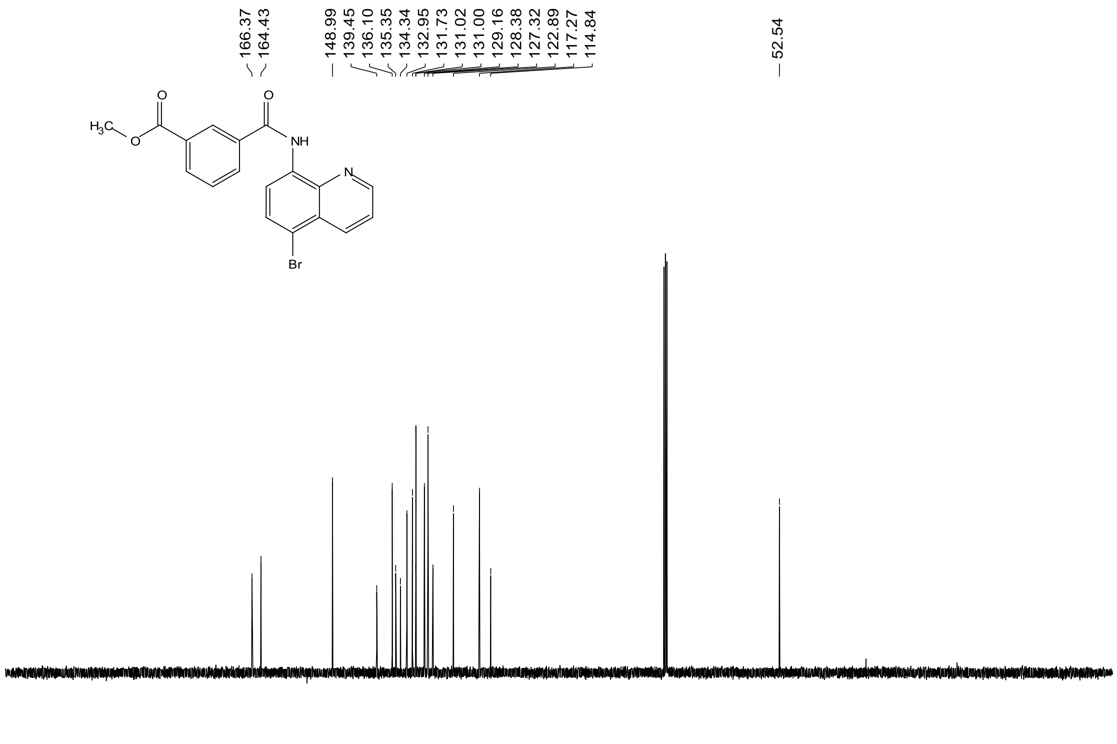

^{1}H NMR for *N*-(5-bromoquinolin-8-yl)-2,4,6-trimethylbenzamide **(3la)**

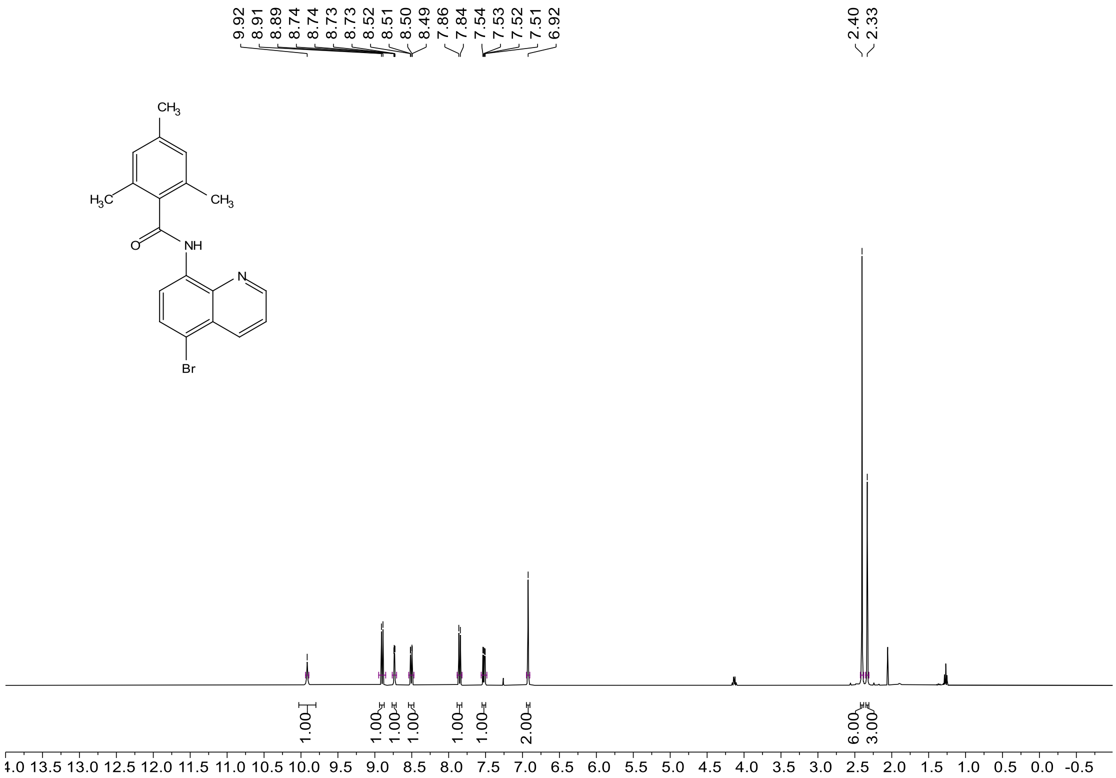

^{13}C NMR for *N*-(5-bromoquinolin-8-yl)-2,4,6-trimethylbenzamide **(3la)**

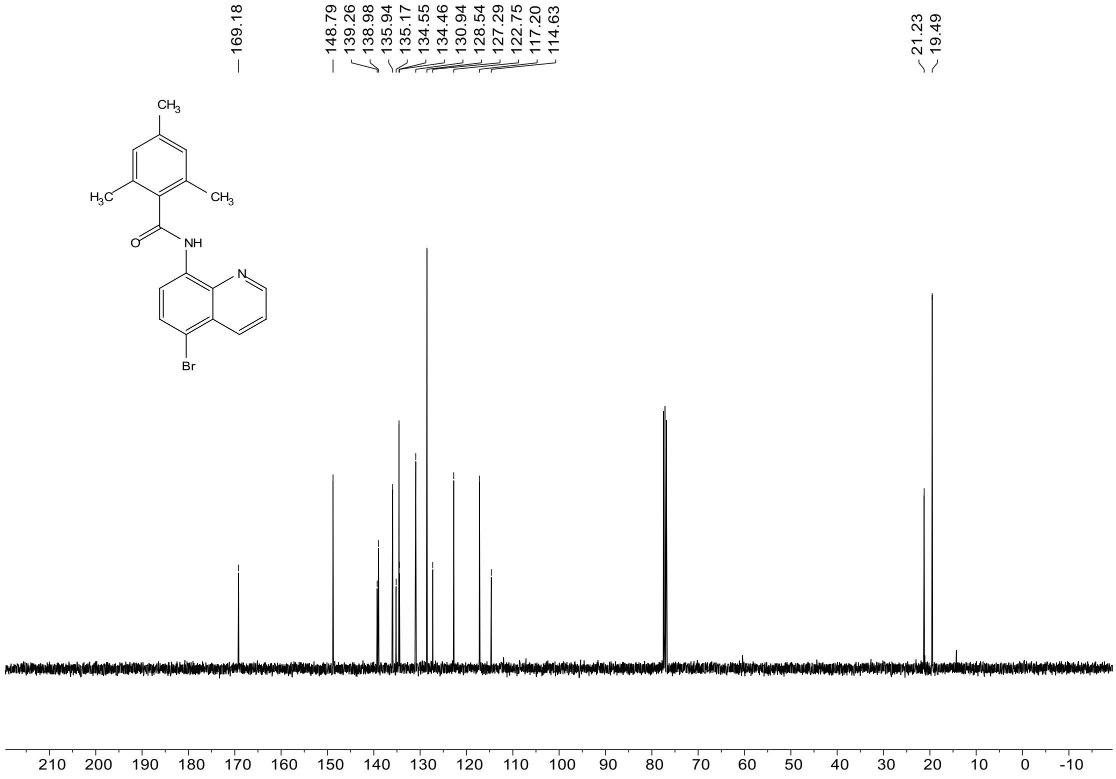

^{1}H NMR for *N*-(5-bromoquinolin-8-yl)-3,4,5-trimethoxybenzamide (**3ma**)

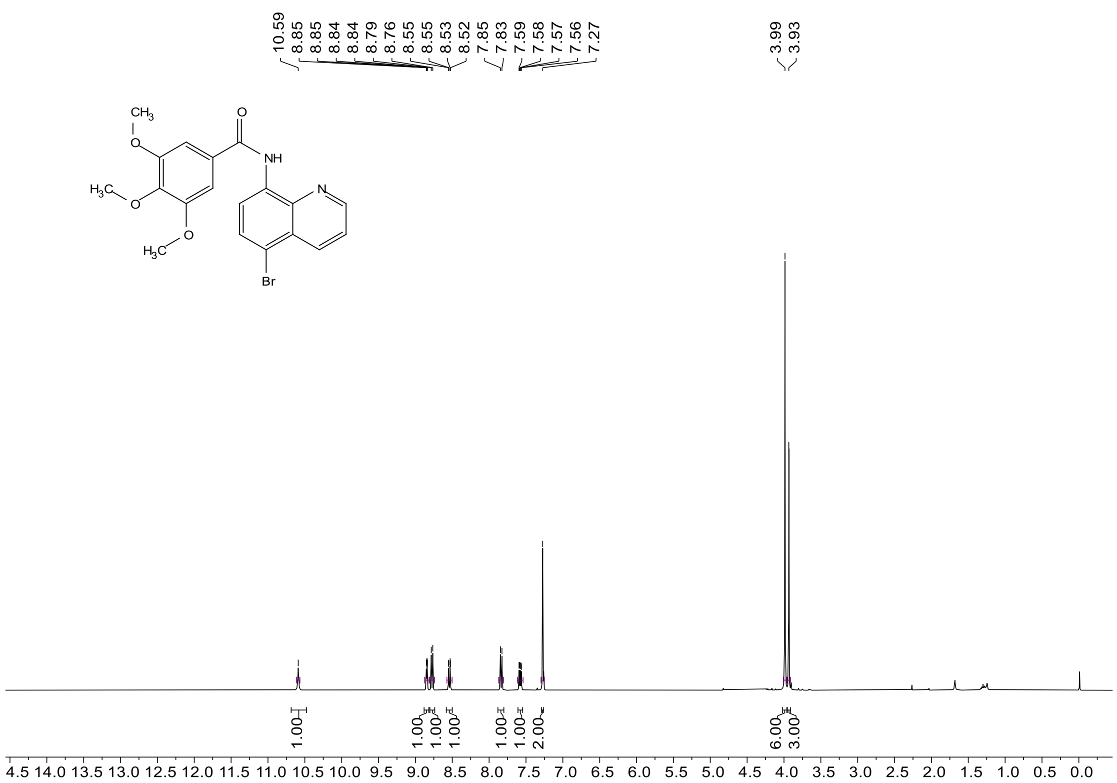

^{13}C NMR for *N*-(5-bromoquinolin-8-yl)-3,4,5-trimethoxybenzamide (**3ma**)

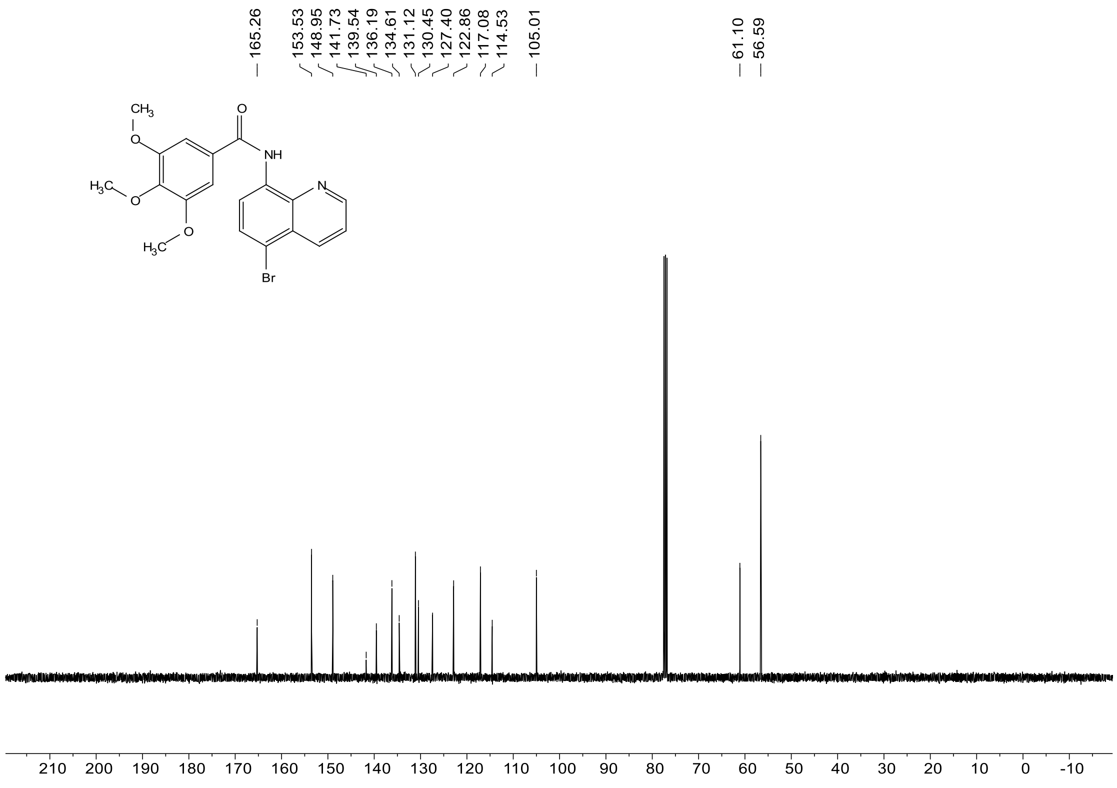

^{1}H NMR for *N*-(5-bromoquinolin-8-yl)-1-naphthamide (**3na**)

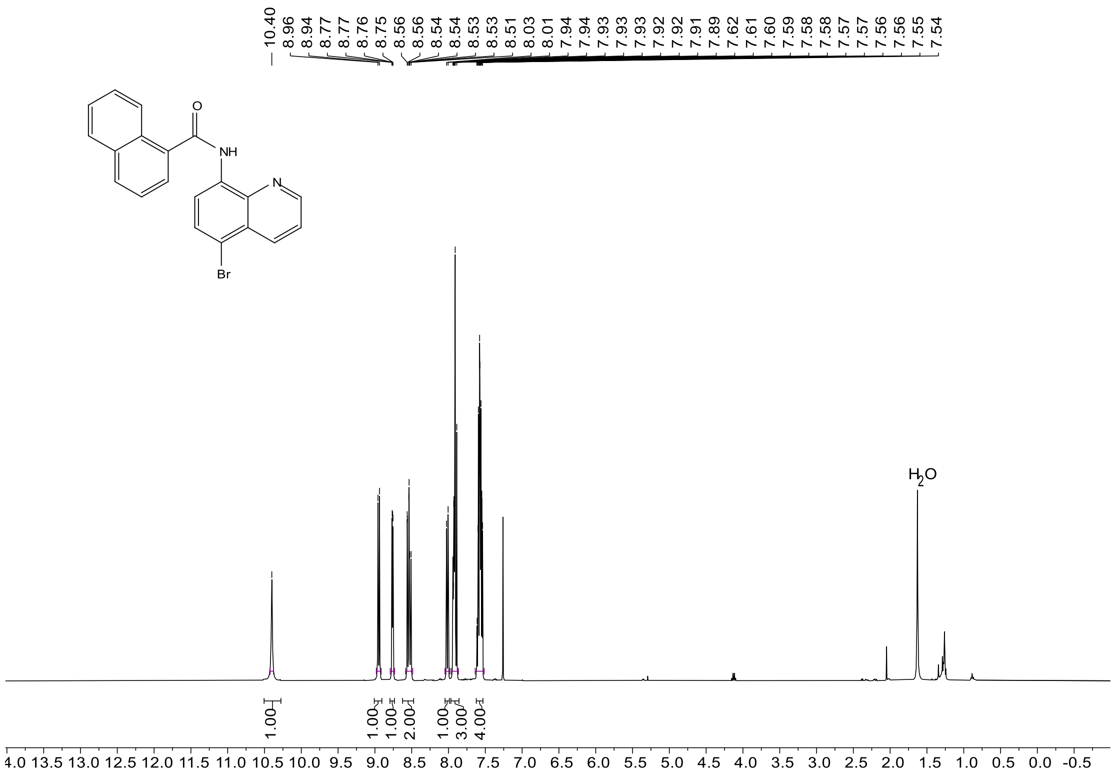

^{13}C NMR for *N*-(5-bromoquinolin-8-yl)-1-naphthamide (**3na**)

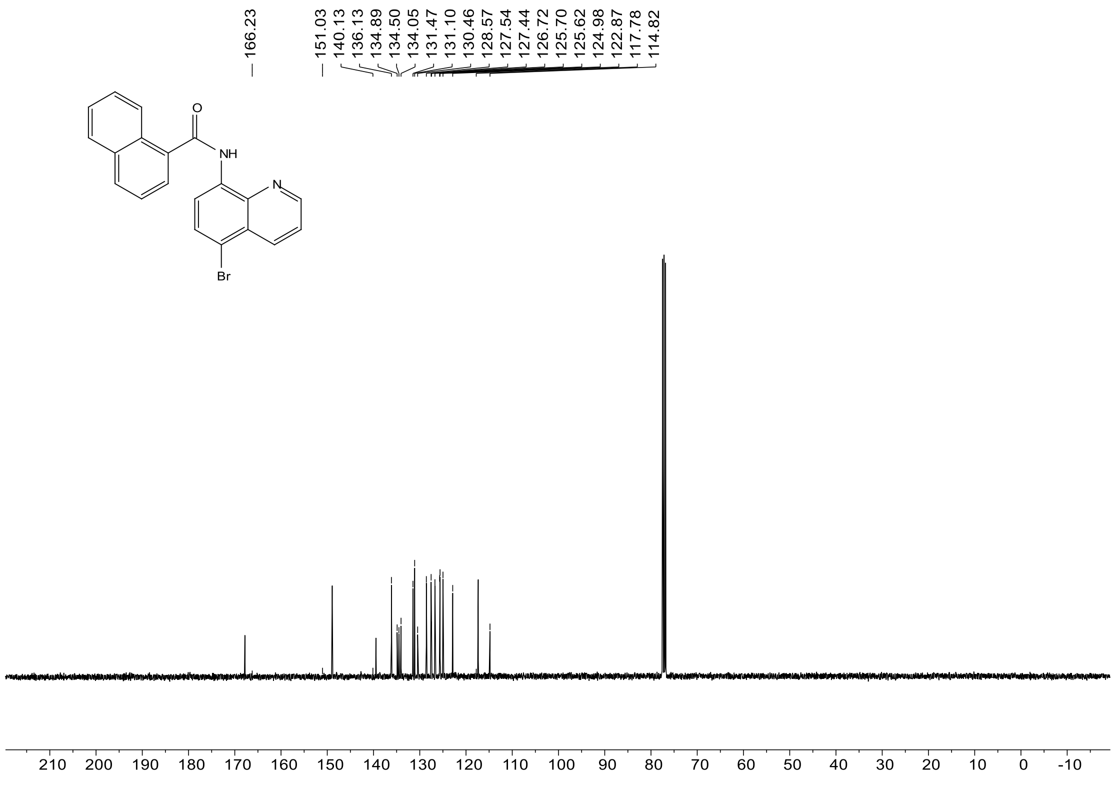

^{1}H NMR for *N*-(5-bromoquinolin-8-yl)furan-2-carboxamide (**3oa**)

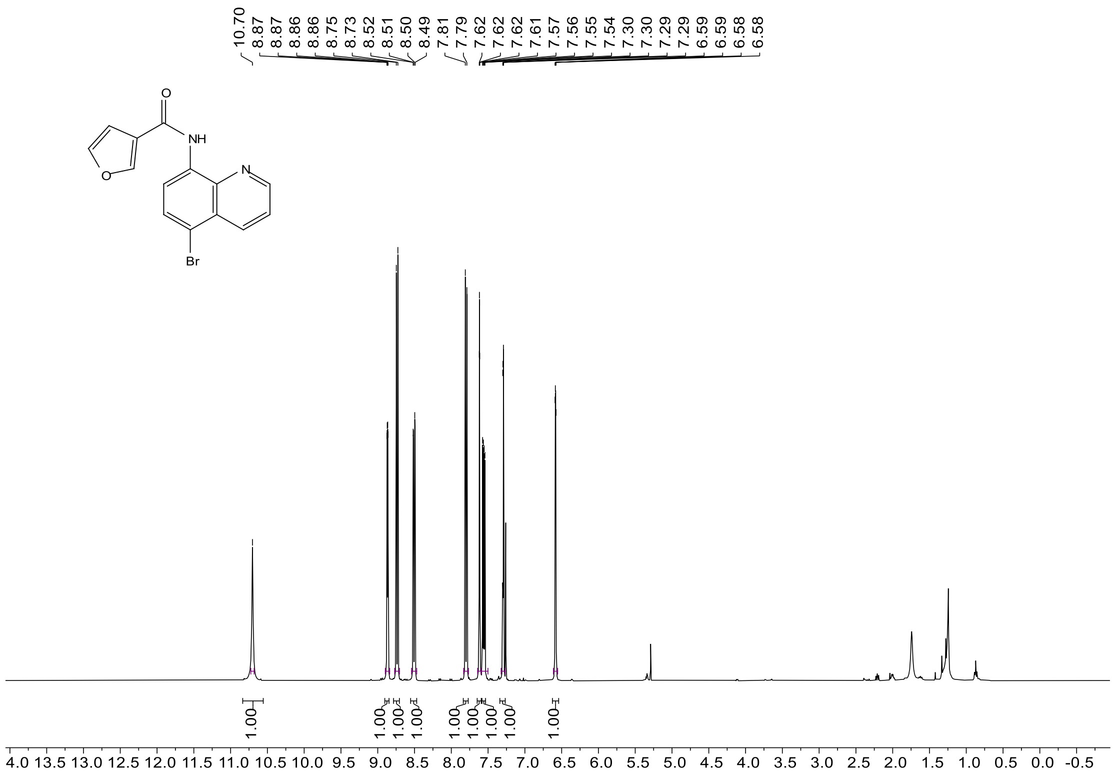

^{13}C NMR for *N*-(5-bromoquinolin-8-yl)furan-2-carboxamide (**3oa**)

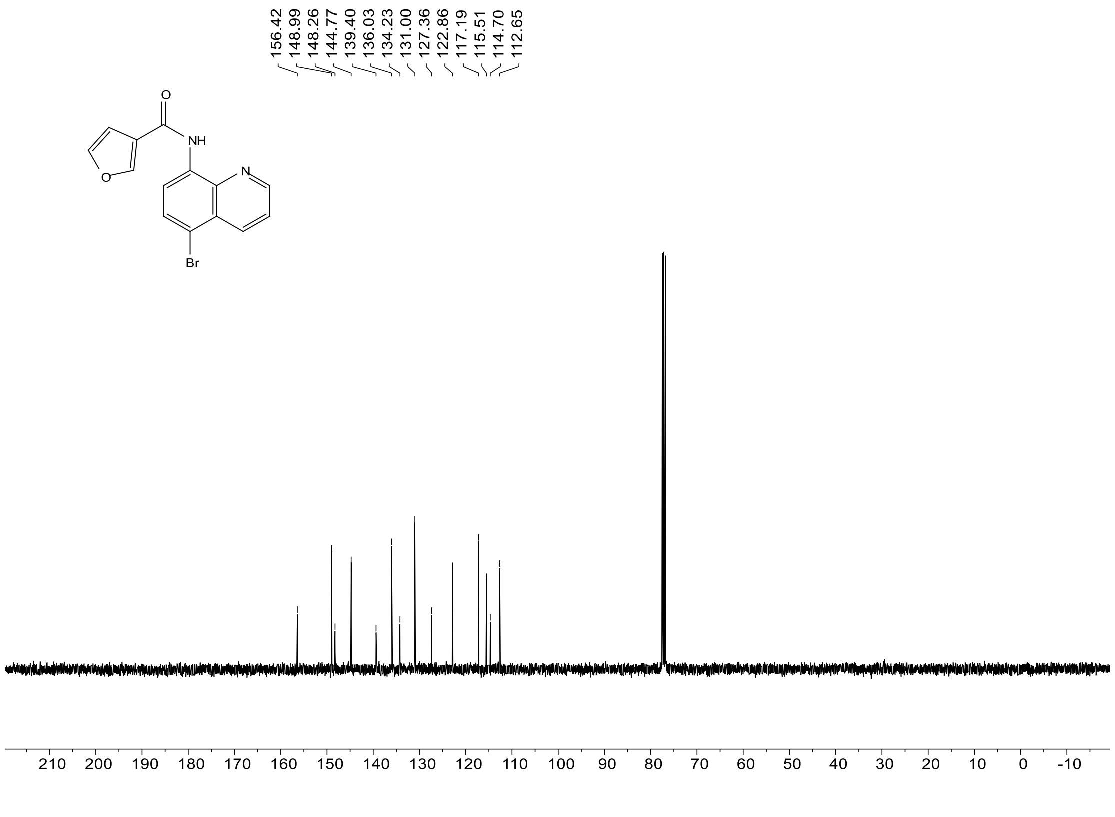

^{1}H NMR for *N*-(5-bromoquinolin-8-yl)thiophene-2-carboxamide (**3pa**)

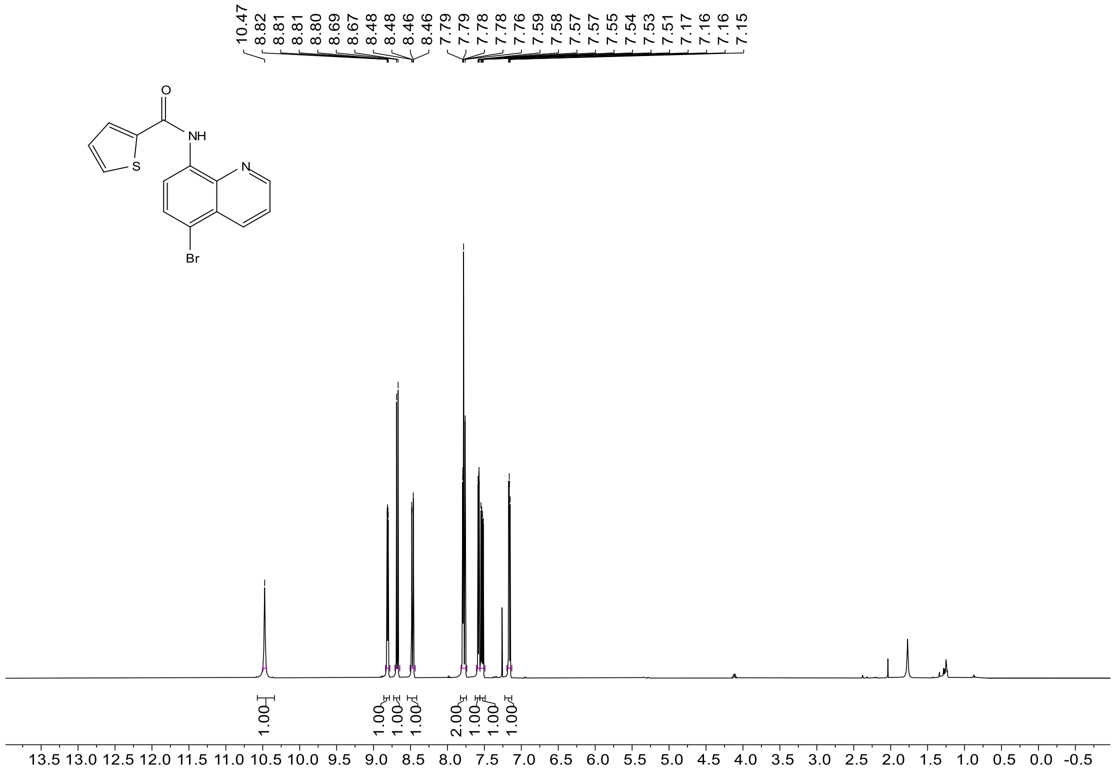

^{13}C NMR for *N*-(5-bromoquinolin-8-yl)thiophene-2-carboxamide (**3pa**)

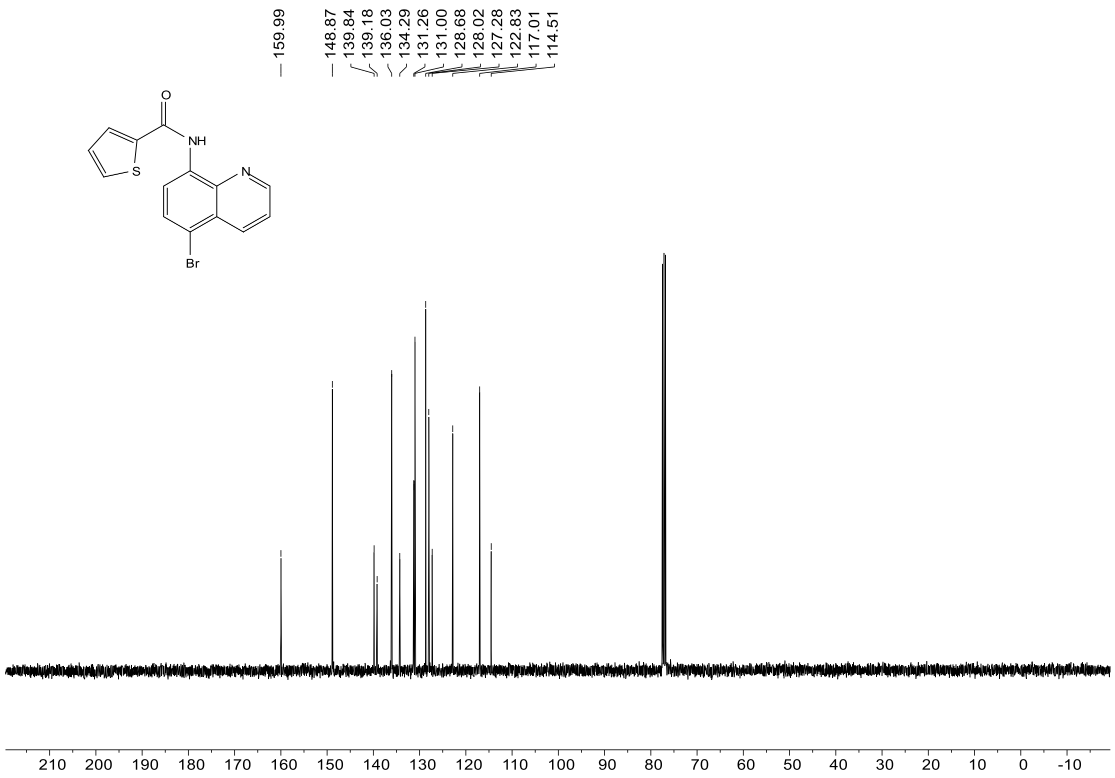

^{1}H NMR for *N*-(5-bromoquinolin-8-yl)-2-phenylacetamide (**3qa**)

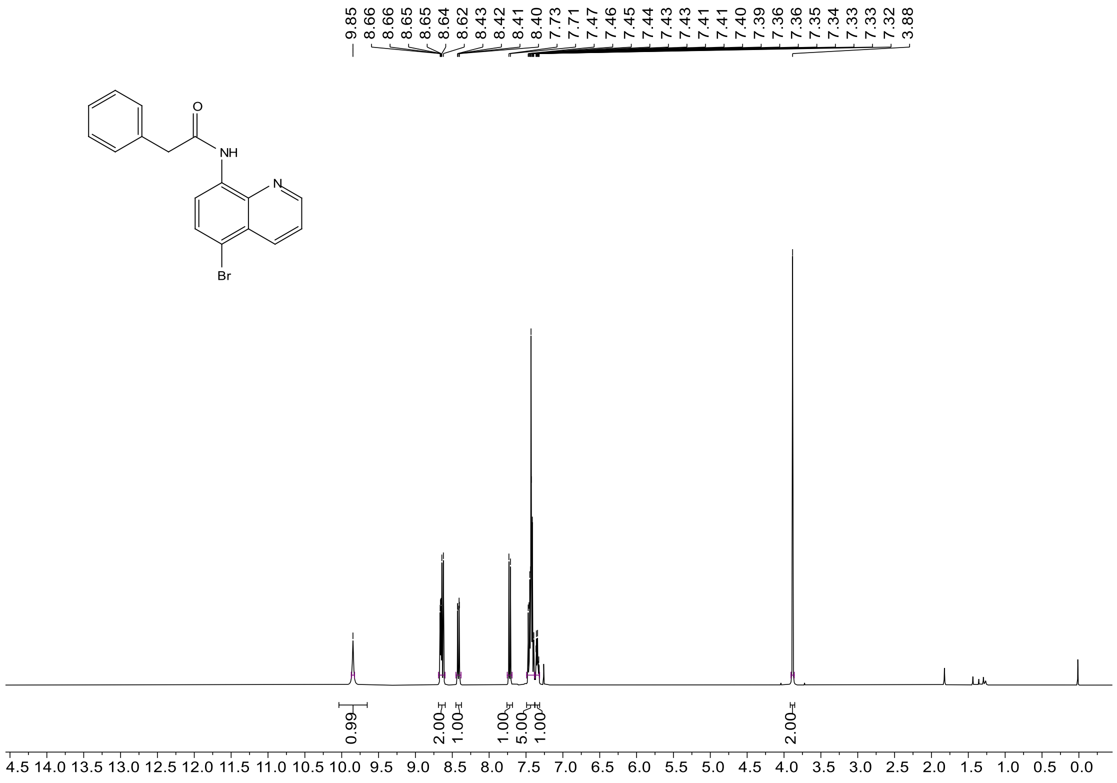

^{13}C NMR for *N*-(5-bromoquinolin-8-yl)-2-phenylacetamide (**3qa**)

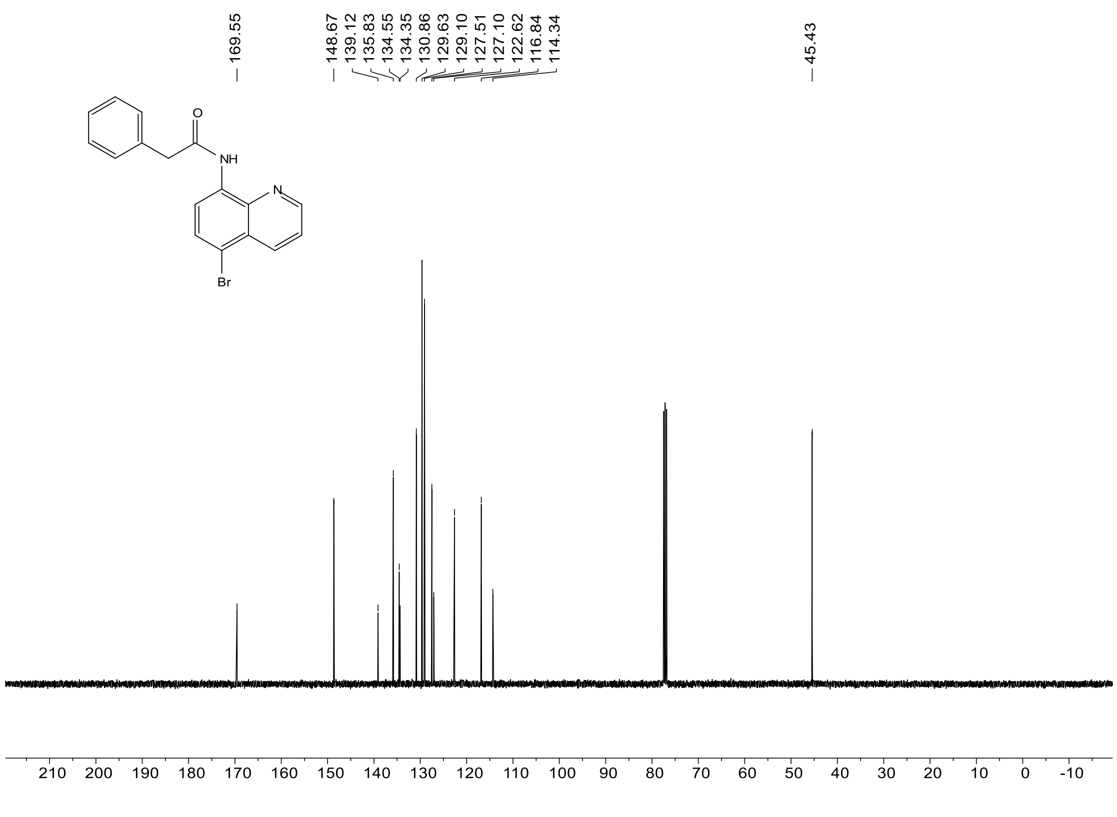

^{1}H NMR for *N*-(5-bromoquinolin-8-yl)acetamide (**3ra**)

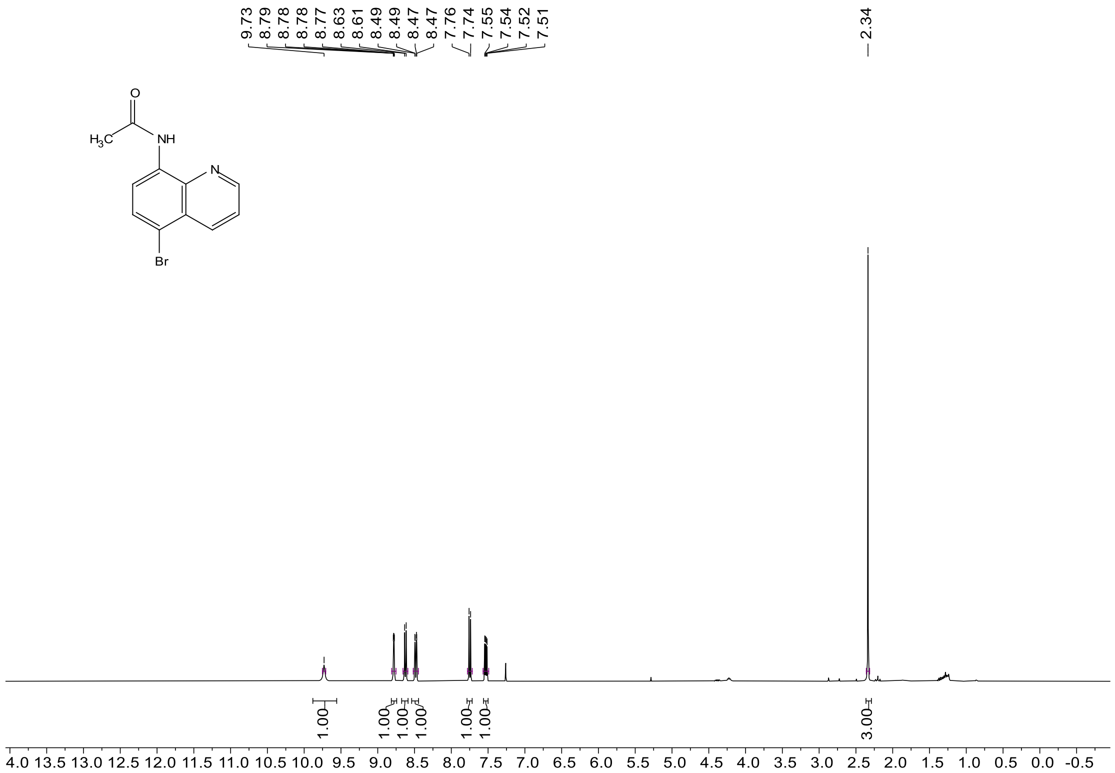

^{13}C NMR for *N*-(5-bromoquinolin-8-yl)acetamide (**3ra**)

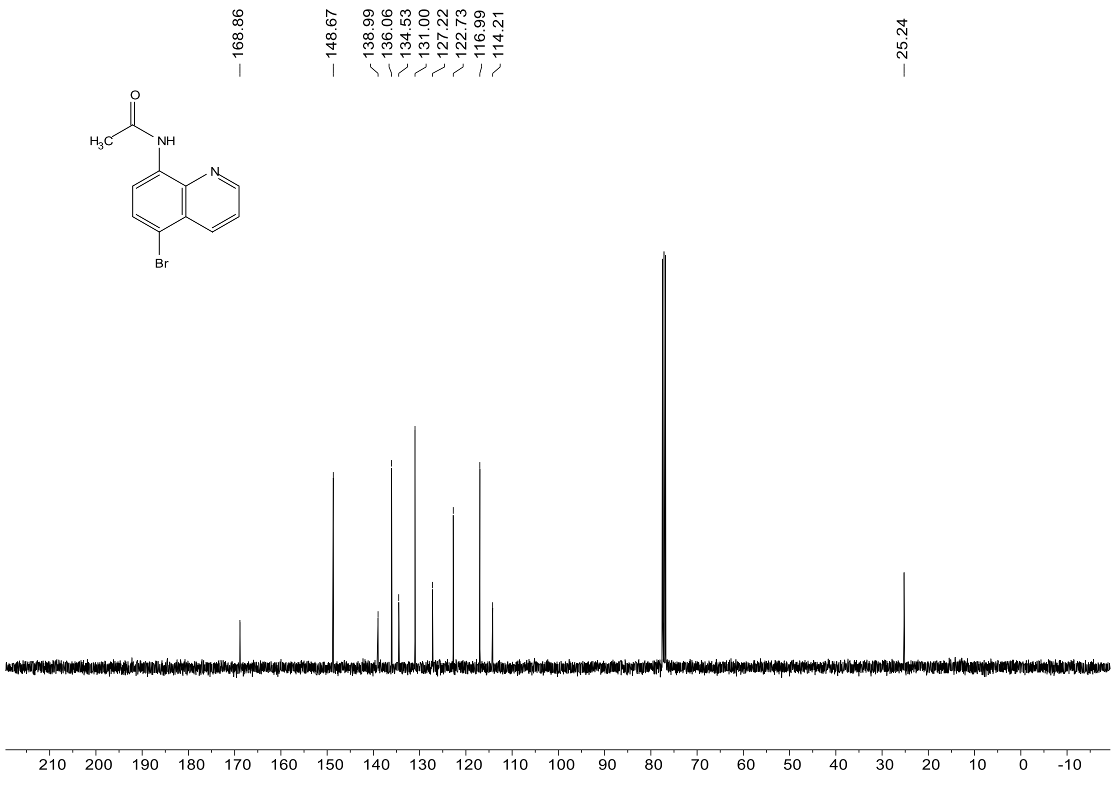

^{1}H NMR for *N*-(5-bromoquinolin-8-yl)isobutyramide (**3sa**)

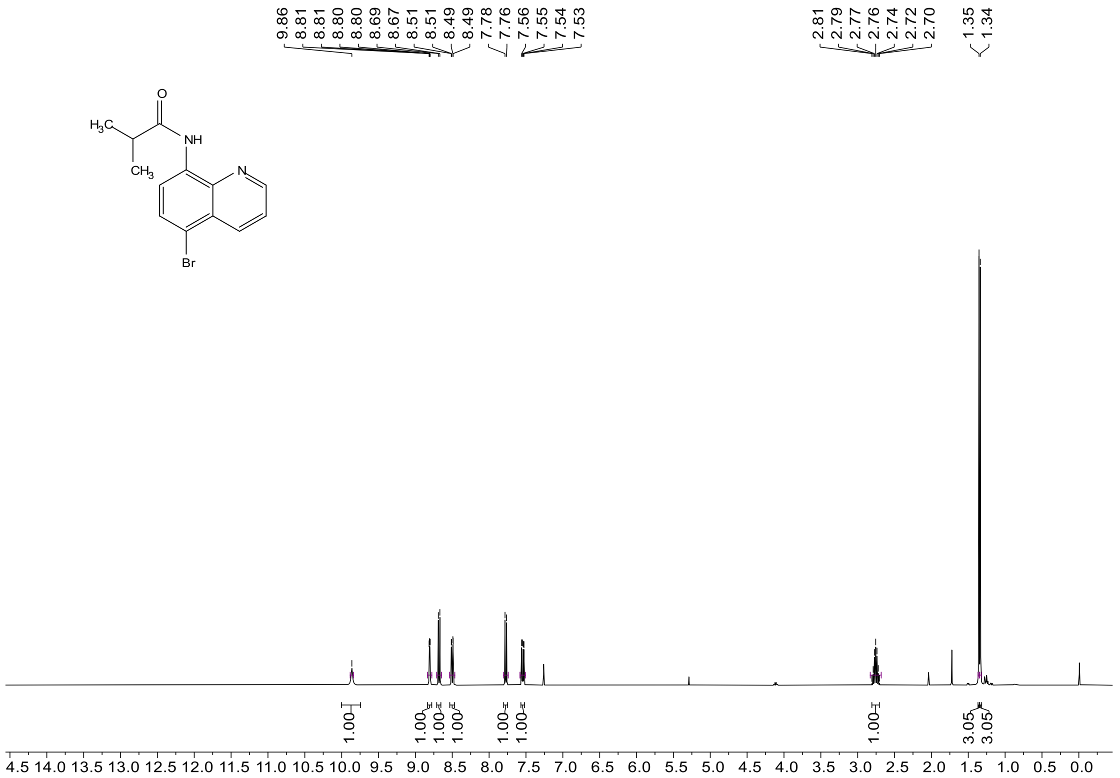

^{13}C NMR for *N*-(5-bromoquinolin-8-yl)isobutyramide (**3sa**)

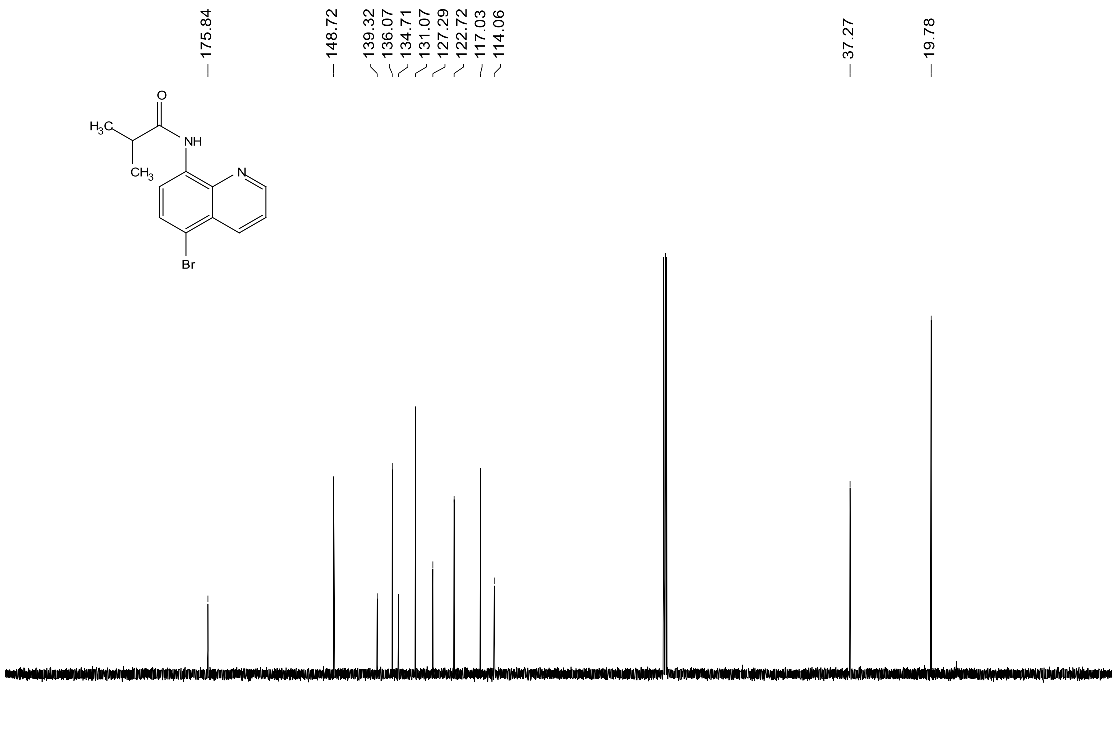

^{1}H NMR for *N*-(5-bromoquinolin-8-yl)pivalamide (**3ta**)

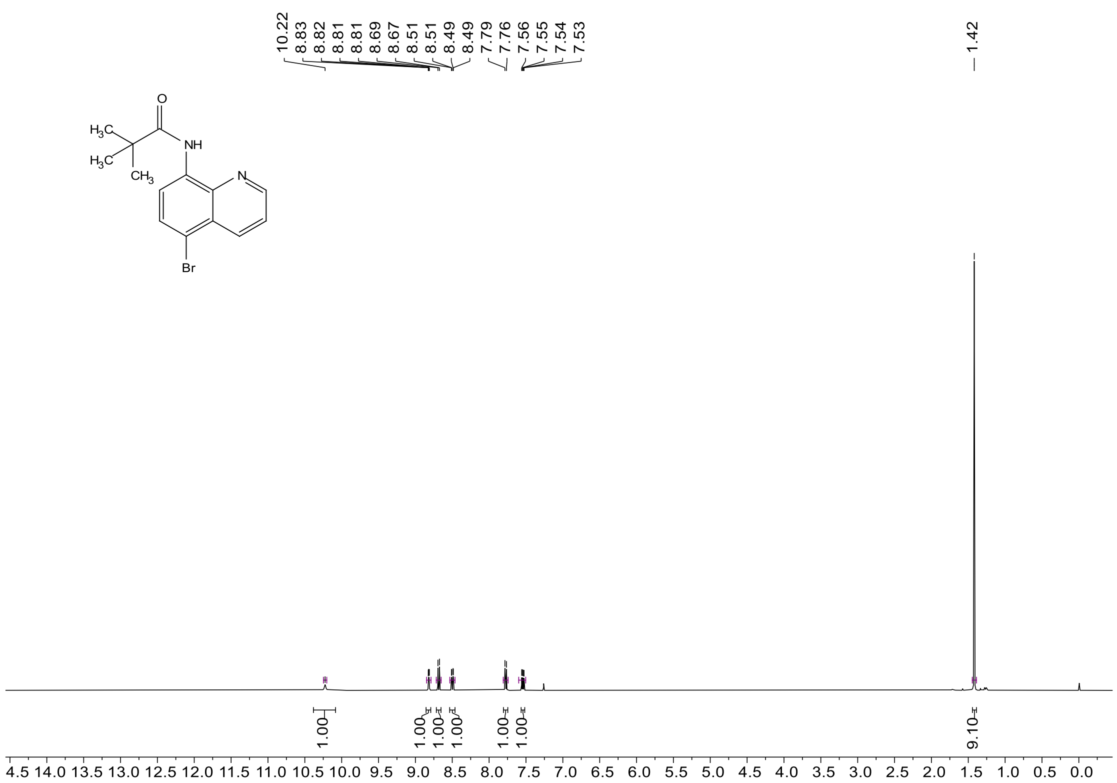

^{13}C NMR for *N*-(5-bromoquinolin-8-yl)pivalamide (**3ta**)

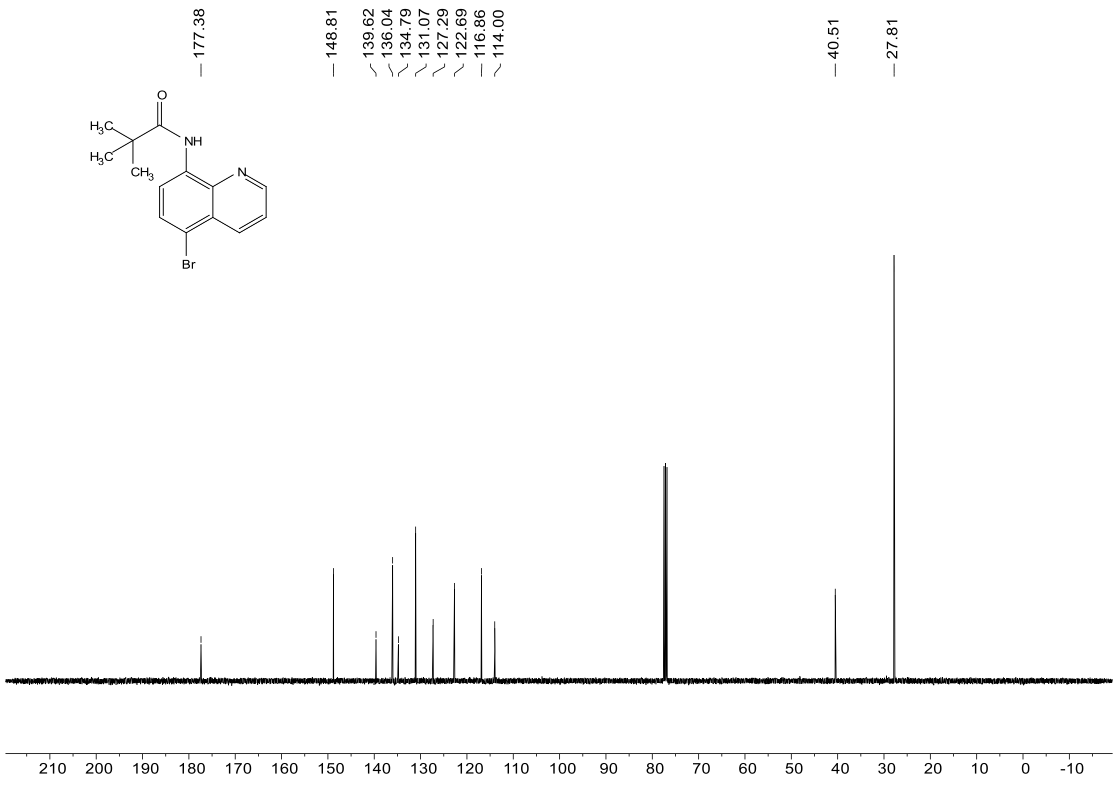

^{1}H NMR for *N*-(5-bromoquinolin-8-yl)cyclopropanecarboxamide (**3ua**)

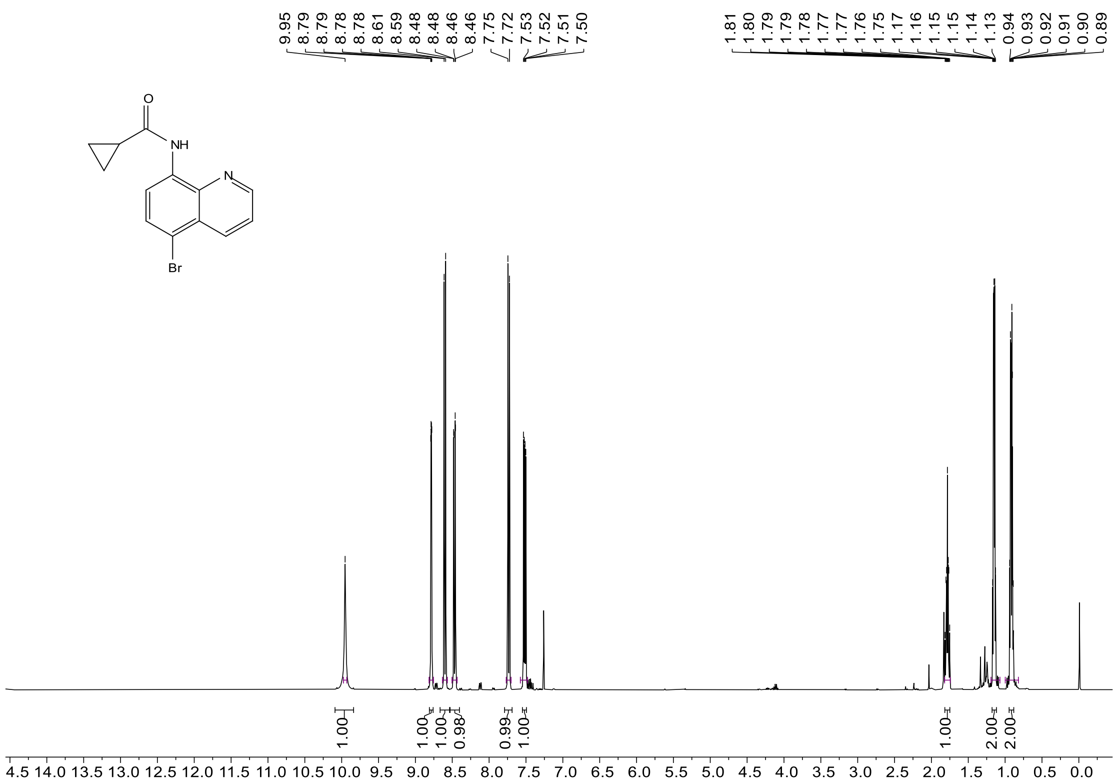

^{13}C NMR for *N*-(5-bromoquinolin-8-yl)cyclopropanecarboxamide (**3ua**)

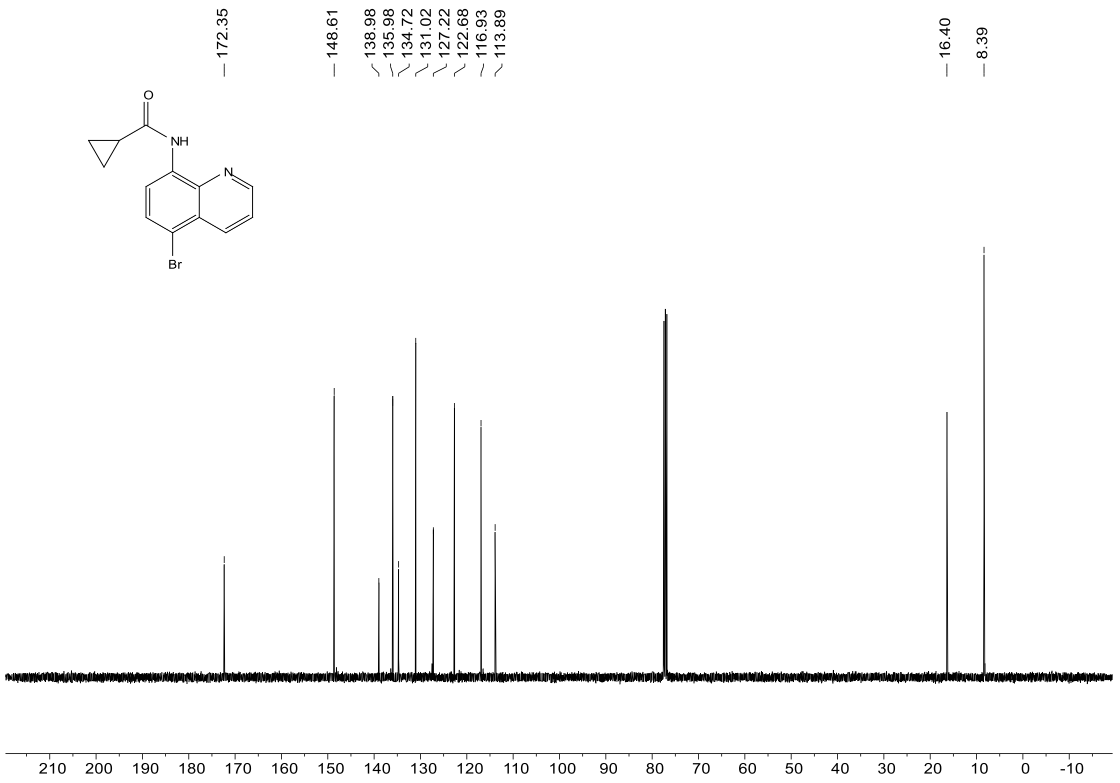

^{1}H NMR for *N*-(5-bromoquinolin-8-yl)cyclobutanecarboxamide (**3va**)

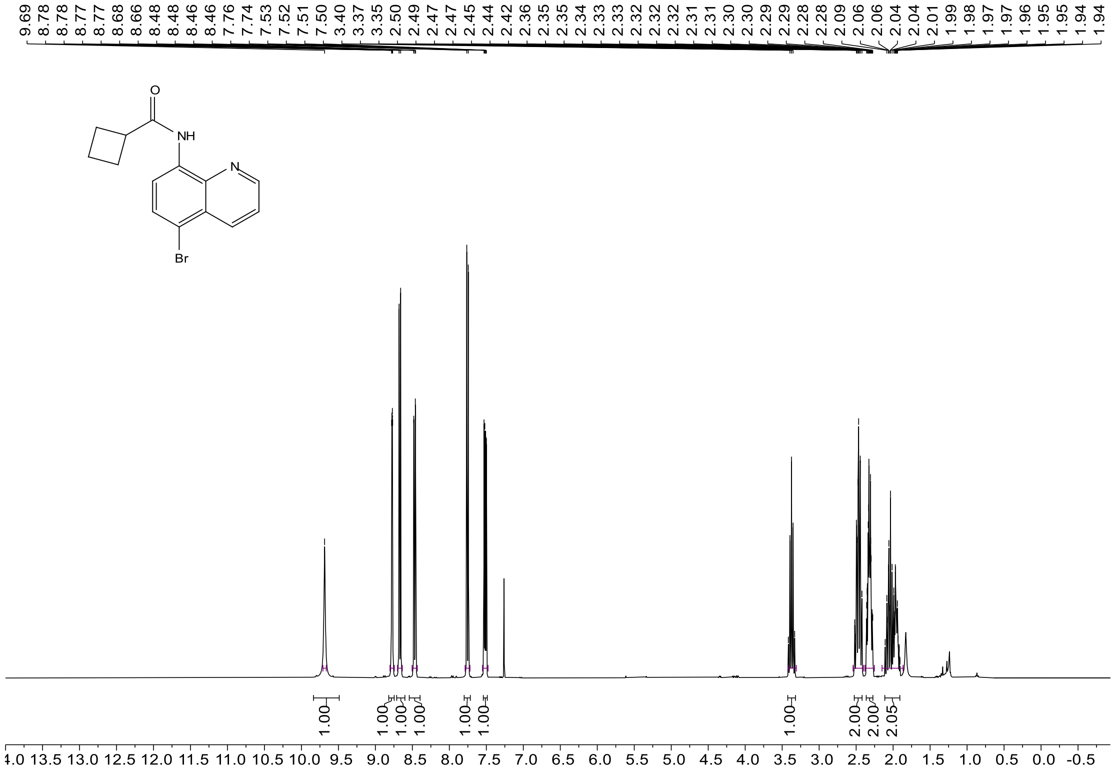

^{13}C NMR for *N*-(5-bromoquinolin-8-yl)cyclobutanecarboxamide (**3va**)

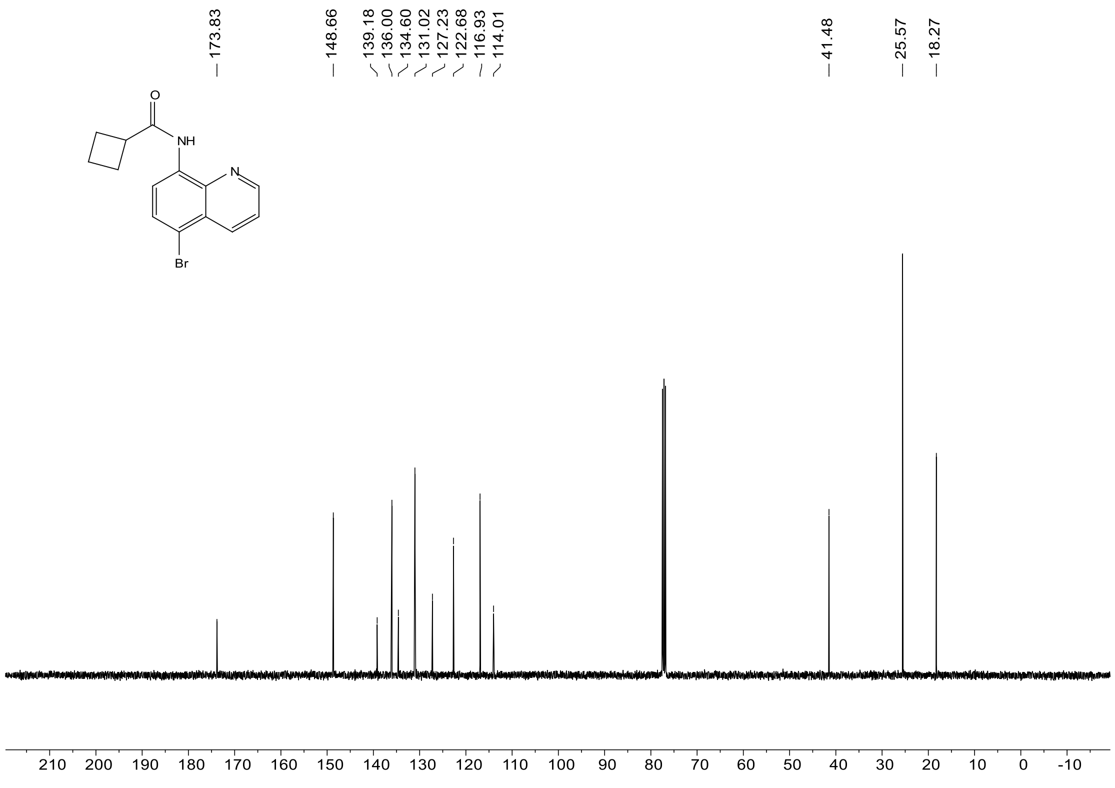

^{1}H NMR for *N*-(5-bromoquinolin-8-yl)-4-methylbenzenesulfonamide (**3za**)

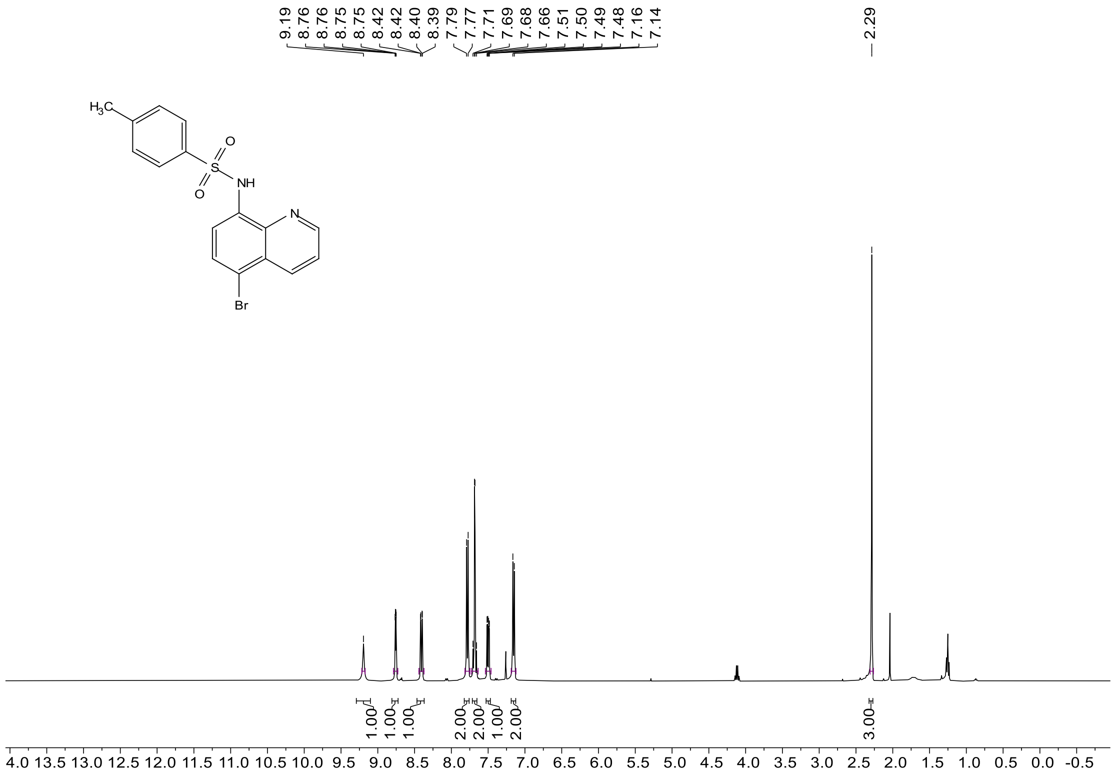

^{13}C NMR for *N*-(5-bromoquinolin-8-yl)-4-methylbenzenesulfonamide (**3za**)

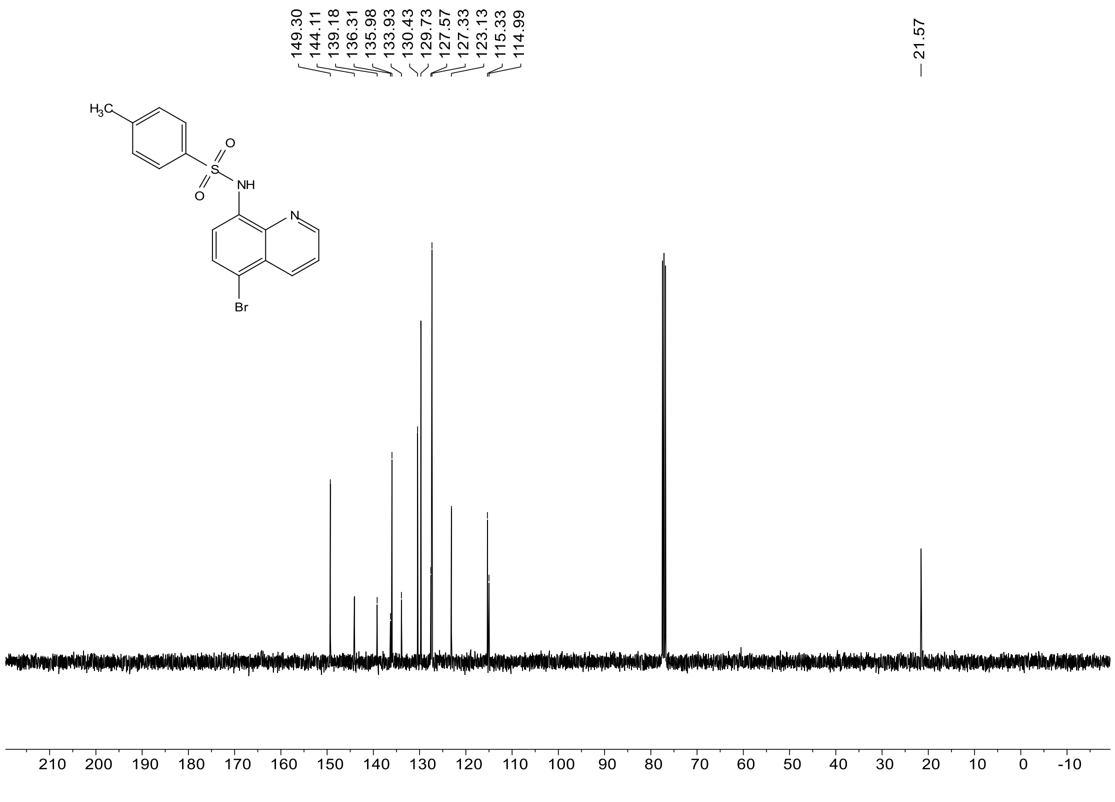

^{1}H NMR for *N*-(5-bromo-6-methoxyquinolin-8-yl)benzamide (**5**)

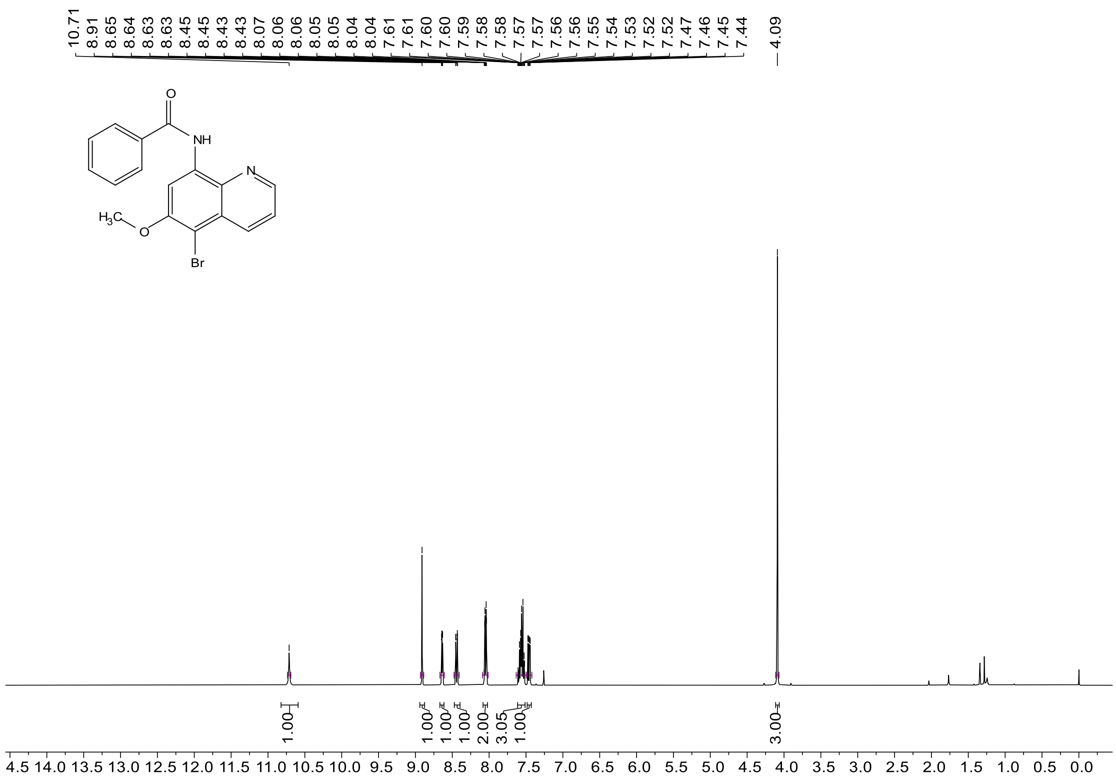

^{13}C NMR for *N*-(5-bromo-6-methoxyquinolin-8-yl)benzamide (**5**)

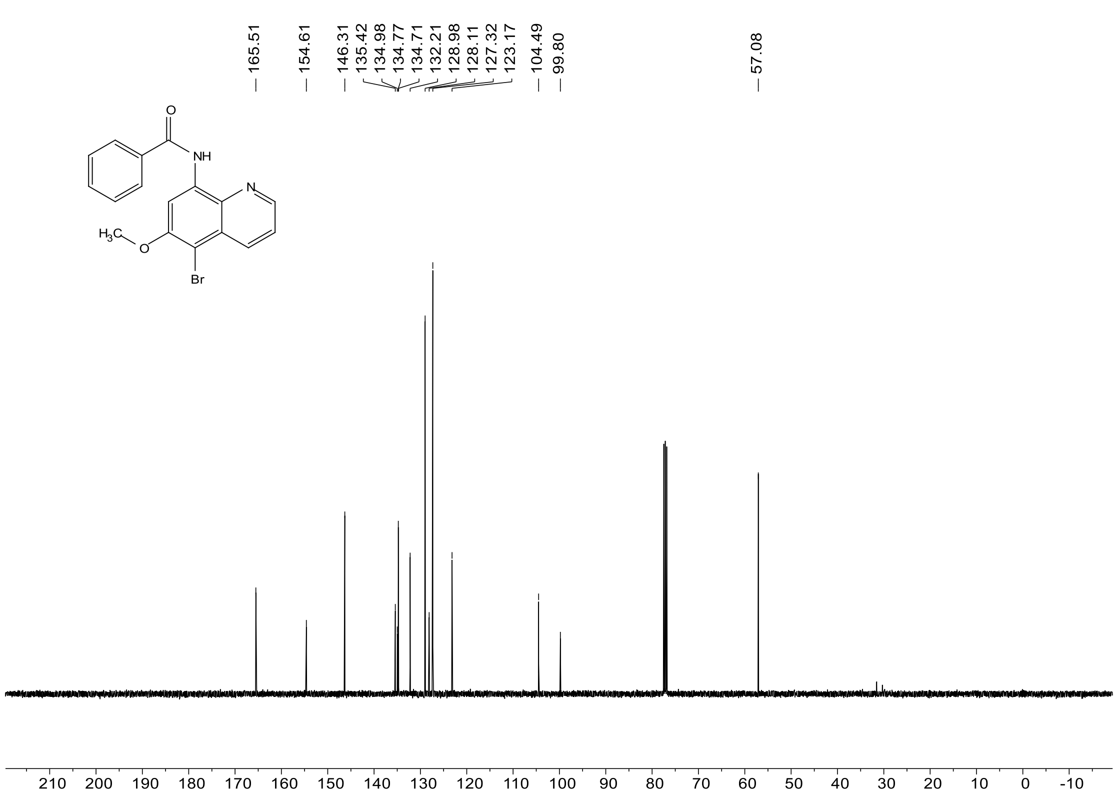

5. References

1. Zhu, L.; Qiu, R.; Cao, X.; Xiao, S.; Xu, X.; Au, C.-T.; Yin, S.-F. *Org. Lett.* **2015**, *17*, 5528–5531. doi:10.1021/acs.orglett.5b02511
2. Kuninobu, Y.; Nishi, M.; Kanai, M. *Org. Biomol. Chem.* **2016**, *14*, 8092–8100. doi:10.1039/c6ob01325b
3. Qiao, H.; Sun, S.; Yang, F.; Zhu, Y.; Kang, J.; Wu, Y.; Wu, Y. *Adv. Synth. Catal.* **2017**, *359*, 1976–1980. doi:10.1002/adsc.201601053
4. Yang, X.; Yang, Q.-L.; Wang, X.-Y.; Xu, H.-H.; Mei, T.-S.; Huang, Y.; Fang, P. *J. Org. Chem.* **2020**, *85*, 3497–3507. doi:10.1021/acs.joc.9b03223
5. Jiao, J.-Y.; Mao, Y.-J.; Feng, A.-W.; Li, X.-F.; Li, M.-T.; Zhang, X.-H. *Tetrahedron* **2017**, *73*, 1482–1488. doi:10.1016/j.tet.2017.01.060
6. Wang, Y.; Wang, Y.; Jiang, K.; Zhang, Q.; Li, D. *Org. Biomol. Chem.* **2016**, *14*, 10180–10184. doi:10.1039/c6ob02079h
7. Guan, Y.; Wang, K.; Shen, J.; Xu, J.; Shen, C.; Zhang, P. *Catal. Lett.* **2017**, *147*, 1574–1580. doi:10.1007/s10562-017-2021-9
8. Shao, C.; Xu, T.; Chen, C.; Yang, Q.; Tang, C.; Chen, P.; Lu, M.; Hu, Z.; Hu, H.; Zhang, T. *RSC Adv.* **2023**, *13*, 6993–6999. doi:10.1039/d3ra00088e
9. Shu, Q.; Li, Y.; Liu, T.; Zhang, S.; Jiang, L.; Jin, K.; Zhang, R.; Duan, C. *Tetrahedron* **2019**, *75*, 3636–3642. doi:10.1016/j.tet.2019.05.037
